# Copper-Catalyzed Synthesis of Unsymmetrical Diorganyl Chalcogenides (Te/Se/S) from Boronic Acids under Solvent-Free Conditions ^‡^

**DOI:** 10.3390/molecules22081367

**Published:** 2017-08-18

**Authors:** Sumbal Saba, Giancarlo Vaccari Botteselle, Marcelo Godoi, Tiago Elias Allievi Frizon, Fábio Zazyki Galetto, Jamal Rafique, Antonio L. Braga

**Affiliations:** 1 Departamento de Química, Universidade Federal de Santa Catarina (UFSC), Florianopolis SC 88040-900, Brazil; sumbal6s@gmail.com (S.S.); galetto.f.z@ufsc.br (F.Z.G.); 2 Department of Chemistry, Shaheed Benazir Bhutto Women University, Peshawar 25000, Pakistan; 3 Laboratório de Pesquisa Química, CECE, Universidade Estadual do Oeste do Paraná UNIOESTE, Toledo PR 85819-110, Brazil; gian.botteselle@gmail.com; 4 Escola de Química e Alimentos, Campus Santo Antônio da Patrulha, Universidade Federal do Rio Grande, Santo Antônio da Patrulha RS 96201-900, Brazil; marcelogodoi@furg.br; 5 Campus Araranguá, Universidade Federal de Santa Catarina (UFSC), Araranguá SC 88905-120, Brazil; tiagofrizon@gmail.com

**Keywords:** selenide, telluride, boronic acid, solvent-free, cross-coupling, CuI, selenium, tellurium

## Abstract

The efficient and mild copper-catalyzed synthesis of unsymmetrical diorganyl chalcogenides under ligand- and solvent-free conditions is described. The cross-coupling reaction was performed using aryl boric acids and 0.5 equiv. of diorganyl dichalcogenides (Te/Se/S) in the presence of 3 mol % of CuI and 3 equiv. of DMSO, under microwave irradiation. This new protocol allowed the preparation of several unsymmetrical diorganyl chalcogenides in good to excellent yields.

## 1. Introduction

Transition metal-catalyzed coupling reactions are among the most commonly applied protocols for the preparation of various target molecules under mild conditions [1,2]. In this regard, the construction of new carbon-carbon and carbon-heteroatom bonds have been described, notably through catalytic processes employing a metal as the catalyst [3,4,5,6,7].

In addition, catalytic transformations have been widely applied in organochalcogen chemistry for the preparation of diorganyl-tellurides, -selenides and -sulfides, which are interesting target molecules and valuable synthetic intermediates for several transformations in modern organic synthesis [8,9,10]. Besides the synthetic applications, these types of molecules have shown relevant biological properties, such as antitumor, antioxidant, antiviral, antimicrobial and neuroprotective effects [11,12,13,14,15,16,17,18,19]. Organochalcogen compounds also have noteworthy applications in materials science [20,21,22].

Due to their considerable importance, the development of new and greener methods for the preparation of organochalcogenides (S, Se and Te) has been widely investigated by several research groups, and a number of methodologies have been reported [23,24,25,26,27,28,29,30]. The most common procedures described for the preparation of alkyl/aryl chalcogenides involve: (a) the reaction between alkyl/aryl dichalcogenide precursors and organometallic reagents [31,32,33,34]; (b) the transition metal-catalyzed reaction of halides with chalcogenols or diorganyl dichalcogenides in the presence of a base or reducing agents [35,36,37]; and (c) synthesis through the C–H activation of arenes [38,39,40,41].

On the other hand, organoboronic acids have been used as suitable reagents for various cross-coupling reactions since they are stable, readily available and compatible with different functional groups. They can also be employed in the boron-to-metal exchange reaction using a catalytic amount of metal [42,43,44]. Thus, organoboronic acids have become interesting and appropriate alternative compounds for the preparation of symmetrical and unsymmetrical diaryl organochalcogenides via cross-coupling transformations.

In this regard, Wang and Tanigushi, independently reported the preparation of diorganyl chalcogenides from organoboronic acids and diorganyl dichalcogenides using copper as a catalyst, in the presence of a ligand and solvent [45,46]. Since their reports, modern studies have been described to improve this kind of transformation. In particular, metals have been efficiently applied as catalysts for the cross-coupling of diorganyl chalcogenides with boronic acids, for instance, employing indium [47], iron [48], copper [49,50,51,52,53,54,55] and silver [56].

However, most of these procedures have their particular drawbacks, such as the use of ligands, reducing agents, air sensitivity, long reaction time, and/or the use of toxic solvents. In addition, some of these methods are not applicable for the synthesis of unsymmetrical organotellurides, which are also very important from the synthetic point of view.

Alternatively, we have recently reported suitable methods for the synthesis of unsymmetrical chalcogenides, using the iodine/DMSO system under microwave irradiation to generate, in situ, electrophilic species of chalcogenyl iodide (RYI) [57]. Similarly, Park and coworkers reported an ultrasound-assisted synthesis of diaryl ditellurides [58]. Nonetheless, the development of new methodologies employing boronic acids and dichalcogenides as starting materials, associated with metal catalysis instead of use RYI species, is still highly desirable. Furthermore, the use of diorganyl dichalcogenides avoids the use of toxic RXH compounds (X = Te, Se, S).

**Scheme 1:**
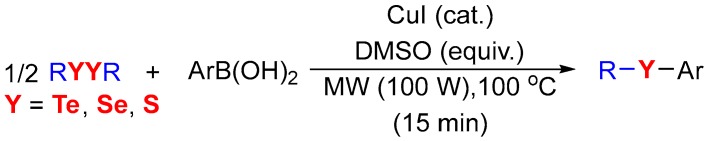
Solvent- and ligand-free synthesis of unsymmetrical diorganyl chalcogenides catalyzed by CuI.

Thus, in connection with our continuing interest in designing and developing eco-friendly processes and cross-coupling reactions, [59,60,61,62,63,64], herein we describe a straightforward method for the synthesis of unsymmetrical diorganyl-chalcogenides (S, Se and Te) through the reaction of boronic acids and half equiv. of diorganyl dichalcogenides, employing CuI as a catalyst, under solvent- and ligand-free conditions (Scheme 1).

## 2. Results and Discussion

In order to optimize the reaction conditions, diphenyl ditelluride (**1a**) and 4-methoxyphenyl boronic acid (**2a**) were used as model substrates (Table 1). Firstly, we evaluated the catalyst loading in the reaction system (entries 1–4). When the reaction was carried out with 1.0 mol % of catalyst the desired product was obtained with only 43% yield (entry 1). However, on increasing the catalyst to 2.0 mol %, the yield of the reaction increased to 71% (entry 2). Notably, when the amount of CuI was increased to 3.0 mol % the corresponding product **3a** was achieved with 90% yield (entry 3). No change in the yield was observed when the catalyst loading was increased to 4.0 mol % (entry 4). 

Next, we investigated the influence of the copper source on the reaction system (entries 5–10). In general, copper halides showed better catalytic activity. For instance, when we employed CuCl_2_ and CuO under the same reaction conditions, the desired organotelluride was synthesized in yields of 72 and 61%, respectively (entries 7 vs. 9). Furthermore, CuO nanoparticles were less effective than copper iodide, affording the desired product in lower yield (entry 10). Considering our previous work [57], when 3% molecular iodine was used as catalyst, **3a** was obtained in 69% yield (entry 11). The reaction in the absence of catalyst provided **3a** in only trace amounts, which emphasizes the notable activity of CuI in this kind of transformation (entry 12).

With the best catalyst in hand, we investigated the influence of other reaction parameters (temperature, time and power) as well as the effect of the additive/oxidant (Table 2).

In this regard, we initially evaluated the influence of a series of additives (CH_3_CN, 1,4-dioxane, EtOH, H_2_O and TBHP). Of these, DMSO was established as the best option, providing the desired product in very high yield in comparison with other additives (entry 1 vs. 2–6). Furthermore, a considerable decrease in the yield was observed when the reaction was carried out either in the absence of an additive or under inert atmosphere, highlighting the need for an oxidant medium for this cross-coupling transformation (entries 7 and 8). In relation to the loading of DMSO, it was observed that 3.0 equiv. was the best alternative, since no improvement in the yield was observed when the amount of this additive was modified (entries 9 and 10).

The influence of the reaction time and temperature was also evaluated (entries 11–14). The screening of these parameters revealed that 15 min and 100 °C were the most appropriate choice for this protocol, as seen in entry 1.

Finally, the effect of the radiation power of the microwave was investigated and increasing the power to 120 W did not change the reaction yield significantly (entry 15). However, when the level of power was decreased to 80 W the desired product **3a** was delivered in lower yield (entry 16). In addition, we carried out the reaction under conventional heating and, even after a long reaction time, a decrease in the yield of the product **3a** was observed (entry 17). This result indicates that microwave irradiation offers advantages over conventional heating, for this transformation.

**Scheme 2:**
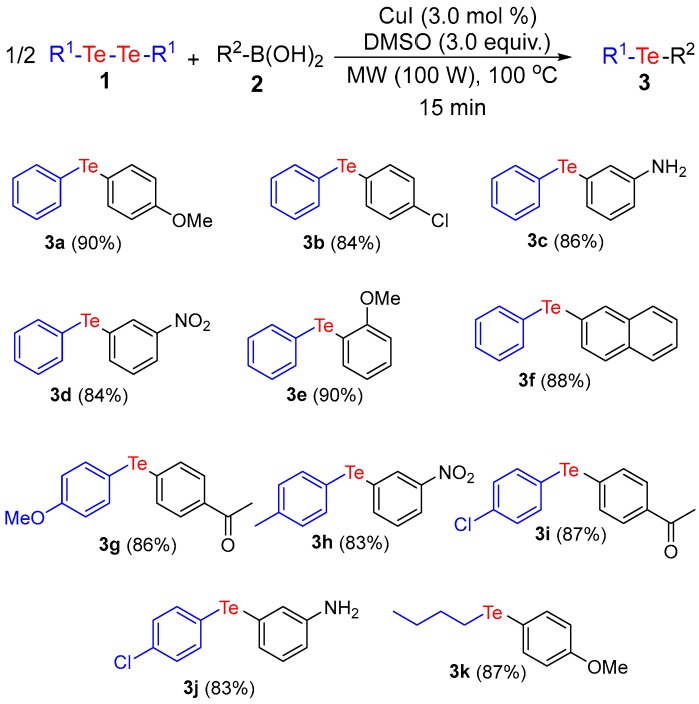
Synthesis of unsymmetrical organotellurides ^a,b^. ^a^ Reaction conditions: **1** (0.25 mmol), **2** (0.5 mmol) in the presence of CuI (3.0 mol %) and DMSO (3.0 equiv.) applied for 15 min at 100 °C with 100 W of MW-irradiation; ^b^ Isolated yields.

Having determined the best reaction conditions, we explored the scope of our protocol by employing various diorganyl ditellurides **1** and arylboronic acids **2** (Scheme 2). Firstly, we evaluated the effect of different groups attached to the aryl ring of boronic acid (Scheme 2).

The electronic characteristics of the substituent of boronic acid attached to the aromatic ring (electron donor or acceptor) and its steric hindrance did not appear to affect the performance of the transformation, and the corresponding products **3a**–**e** were obtained in very good yields. Also, 2-naphthylboronic acid reacted very well with diphenyl ditelluride (**1a**) under the optimized conditions, affording the corresponding product **3f** in very good yield.

We further evaluated the generality of the reaction regarding the ditellurides **1**, employing electron-withdrawing or electron-donating groups. In general, the reaction proceeded very well in both cases, furnishing the corresponding unsymmetrical tellurides **3g**–**j** in 83–87% yields. For instance, the reaction of *para*-chlorobenzene ditelluride with *meta*-substituted aryl boronic acid delivered the respective compound **3j** in 83% yield. Moreover, an aliphatic ditelluride, in this case dibutyl ditelluride, was also a suitable substrate for this cross-coupling process, furnishing the product **3k** in 87% yield.

**Scheme 3:**
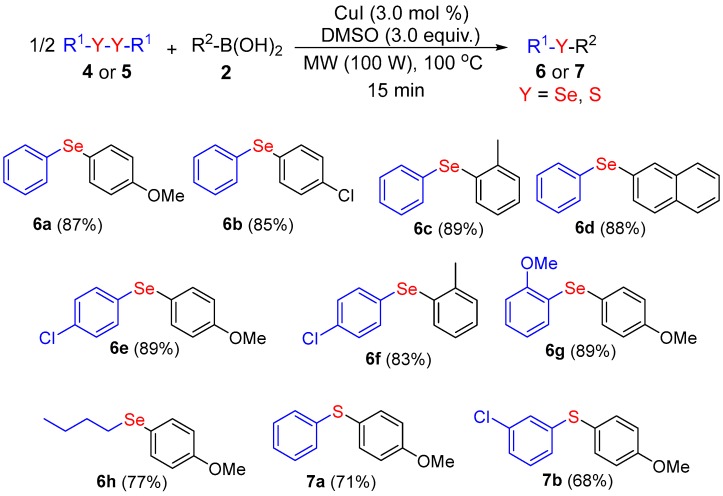
Synthesis of unsymetrical organoselenides and sulfides ^a,b^. ^a^ Reaction conditions: **4** or **5** (0.25 mmol), **2** (0.5 mmol) in the presence of CuI (3.0 mol %) and DMSO (3.0 equiv.) for 15 min at 100 °C and 100 W of MW-irradiations; ^b^ Isolated yields.

The successful preparation of unsymmetrical diorganyl tellurides **3** by a copper-catalyzed transformation prompted us to expand this approach to the synthesis of unsymmetrical diorganyl selenides (Scheme 3). To evaluate the electronic effects, we first verified the influence of the substituent attached at the *para* position of the aromatic ring of the boronic acid. The treatment of diphenyl diselenide with different *para*-substituted aryl boronic acids provided the respective products **6a** and **6b** in 87% and 85% yields, respectively.

Similarly, the reaction was not sensitive to steric effects, since the treatment of diphenyl diselenide with aryl boronic acid containing a methyl group at the *ortho* position of the aromatic ring provided the respective product **6c** in 89% yield. Subsequently, a series of different diselenides, including aliphatic and aromatics diselenides, were converted into the corresponding products in very good yields. For example, when we reacted *p*-chlorobenzene diselenide with 4-methoxyphenylboronic acid the product **6e** was obtained in 89% yield.

We also evaluated the applicability of our protocol to the preparation of unsymmetrical sulfides, under the same reaction conditions. Remarkably, diphenyl disulfide and *m*-chlorophenyl disulfide reacted smoothly with *p*-methoxyphenyl boronic acid, leading to the respective products **7a** and **7b** in 71% and 68% yields, respectively. This slight decrease in the yield values could be associated with the lower reactivity of diaryl disulfides when compared to ditellurides or diselenides analogues, which are much more easily cleaved than disulfides [65].

**Scheme 4:**
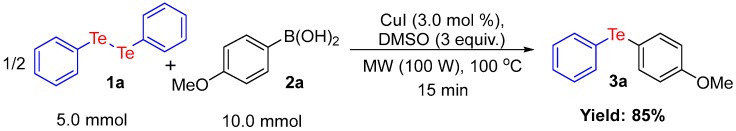
Scale-Up of the reaction.

In addition, in order to investigate the synthetic utility of this methodology, we evaluated whether the reaction could be performed with boronic acid at the gram scale under the optimized conditions (Scheme 4). To our delight, the corresponding product **3a** was obtained in 85% yield, which is very significant from a synthesis point of view, since this methodology can thus be used to prepare unsymmetrical diorganyl chalcogenides on a larger scale.

**Scheme 5:**
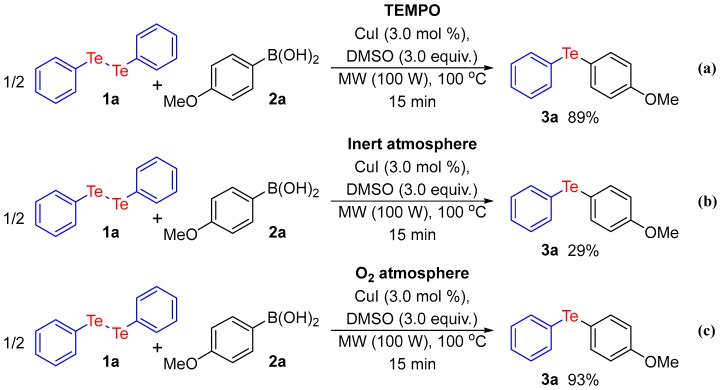
Control experiments for reaction mechanism. (**a**) Reaction in the presence of radical inhibitor; (**b**) Reaction under inert atmosphere; (**c**) Reaction under oxygen atmosphere.

In order to gain further insight into the reaction mechanism, control experiments were performed (Scheme 5). Firstly, to evaluate the possibility of a radical path, we performed the standard reaction in the presence of 3.0 equiv. of 2,2,6,6-tetramethyl-1-piperidinyloxy (TEMPO) as a radical inhibitor (Scheme 5a). The presence of TEMPO did not affect the reaction yield, which suggests that radical intermediates are not involved in this protocol. Next, we verified the influence of oxidant species on this transformation. When the reaction was carried out under argon atmosphere, an abrupt decrease was noted and the telluride **3a** was delivered in only 29% yield (Scheme 5b). In contrast, when diphenyl ditelluride was treated with phenyl boronic under O_2_ atmosphere, the corresponding product was obtained in 93% yield (Scheme 5c). These results suggest that oxygen is mainly responsible for the oxidation steps and DMSO is most likely an important additive in terms of improving the reaction yield.

**Scheme 6:**
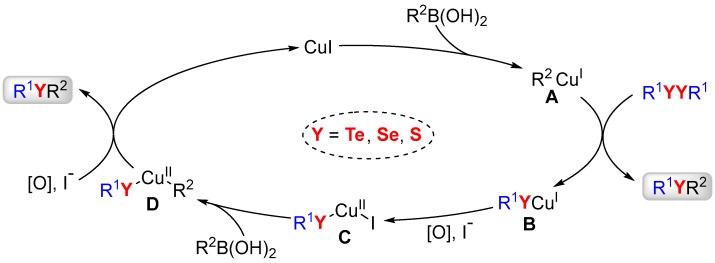
A plausible reaction pathway.

On the basis of these results and in accordance with previous reports [66,67], a plausible reaction pathway is illustrated in Scheme 6. In this pathway the catalyst cycle begins with the generation of species **A** through the reaction between CuI and boronic acid [66]. Subsequently, this specie reacts with diorganyl dichalcogenide to afford the desired product and the intermediate **B**. The specie **B** is then oxidized to give the Cu(II) intermediate **C** [66,67]. In the next step, another equiv. of boronic acid would reacts with this intermediate to furnish the intermediate **D**. Lastly, a second equiv. of the desired product is obtained through an elimination reaction step and, consequently, the catalyst is regenerated.

## 3. Materials and Methods

### 3.1. General Information

^1^H- and ^13^C-NMR spectra were obtained at 200/50 MHz on an AC-200 NMR spectrometer (Bruker, Rheinstetten, Germany) or at 400/100 MHz on an AS-400 NMR spectrometer (Varian, Palo Alto, CA, USA). Spectra were recorded in CDCl_3_ solutions. Chemical shifts are reported in ppm, referenced to the solvent peak of CDCl_3_ or tetramethylsilane (TMS) as the external reference. Data are reported as follows: Chemical shift (δ), multiplicity, coupling constant (*J*) in hertz and integrated intensity. Abbreviations to denote the multiplicity of a particular signal are: s (singlet), d (doublet), t (triplet), q (quartet), quint (quintet), sext (sextet) and m (multiplet). The NMR spectra are found in the Supplementary Materials. The melting points were determined using a model MQRPF-301 digital apparatus (Microquimica, Palhoça, Brazil) equipped with a heating plate. Column chromatography was performed using silica gel (230–400 mesh). Thin layer chromatography (TLC) was performed using silica gel GF_254_ plates with 0.25 mm thickness (Merck, Darmstadt, Germany). For visualization, TLC plates were either placed under ultraviolet light or stained with iodine vapor and acidic vanillin. Most reactions were monitored by TLC for the disappearance of the starting material.

Unless otherwise stated, all reactions were carried out in an open atmosphere. All reagents and solvents were obtained from commercial sources and used without further purification. All reactions were performed in 10 mL sealed glass tubes in a commercially available monomode microwave reactor (CEM, Matthews, NC, USA) with IR monitoring and a non-invasive pressure transducer. Reagents and solvents were handled using standard syringe techniques. The yields are based on isolated compounds after purification.

### 3.2. General Procedure for the Synthesis of Unsymmetrical Organochalcogenides under MW Irradiation

A mixture of aryl boronic acid (0.5 mmol), dichalcogenide (0.25 mmol), CuI (3 mol %, 1.5 mg), and DMSO (3 equiv., 59 mg) was placed in a glass tube. The tube was sealed with a pressure lock and the mixture was heated in air at 100 °C for 15 min with the aid of an initial MW irradiation of 100 W in a CEM Discover MW reactor. When the reaction was finished, the crude mixture was cooled to room temperature, diluted with ethyl acetate (10 mL), and washed with water (3 × 5 mL). The organic phase was separated, dried over MgSO_4_, and concentrated under vacuum. The residue was purified by flash chromatography on silica gel using hexane or a mixture of ethyl hexane/acetate (99:1) as the eluent.

#### Analytical Data of Products **3a**–**k**, **6a**–**h** and **7a**,**b**

*(4-Methoxyphenyl)(phenyl)tellane* (**3a**). Yield: 0.140 g (90%); white solid; m.p.: 59–61 °C (lit.: [48] 60–62 °C); ^1^H-NMR (200 MHz, CDCl_3_, ppm) δ = 7.73 (d, *J* = 8.8 Hz, 2H, H-Ar), 7.62–7.42 (m, 2H, H-Ar), 7.30–7.07 (m, 3H, H-Ar), 6.80 (d, *J* = 8.8 Hz, 2H, H-Ar), 3.80 (s, 3H, -OCH_3_); ^13^C-NMR (50 MHz, CDCl_3_, ppm) δ = 160.1, 141.3, 136.5, 129.4, 127.4, 116.0, 115.6, 103.3, 55.3.

*(4-Chlorophenyl)(phenyl)tellane* (**3b**). Yield: 0.133 g (84%); yellow oil; [68] ^1^H-NMR (200 MHz, CDCl_3_, ppm) δ = 7.63–7.57 (m, 2H, H-Ar), 7.52–7.46 (m, 2H, H-Ar), 7.21–7.05 (m, 5H, H-Ar); ^13^C-NMR (50 MHz, CDCl_3_, ppm) δ = 139.3, 138.2, 134.4, 129.8, 129.7, 128.2, 114.5, 112.5.

*3-(Phenyltellanyl)aniline* (**3c**). Yield: 0.128 g (86%); yellow oil; [57] ^1^H-NMR (200 MHz, CDCl_3_, ppm) δ *=* 7.57 (d, *J* = 8.4 Hz, 2H, H-Ar), 7.56–6.99 (m, 6H, H-Ar), 6.60–6.55 (m, 1H, H-Ar), 3.62 (s, 2H, -NH_2_); ^13^C-NMR (50 MHz, CDCl_3_, ppm) δ = 147.3, 138.0, 130.3, 129.6, 128.2, 127.8, 124.4, 115.4, 114.9, 114.8.

*(3-Nitrophenyl)(phenyl)tellane* (**3d**). Yield: 0.137 g (84%); yellow oil; [55] ^1^H-NMR (400 MHz, CDCl_3_, ppm) δ = 8.42 (s, 1H, H-Ar), 8.06 (d, *J* = 8.2 Hz, 1H, H-Ar), 7.86–7.80 (m, 3H, H-Ar), 7.41–7.25 (m, 4H, H-Ar); ^13^C-NMR (101 MHz, CDCl_3_, ppm) δ = 148.5, 142.4, 139.6, 131.1, 130.1, 130.0, 129.1, 122.5, 117.0, 113.2.

*(2-Methoxyphenyl)(phenyl)tellane* (**3e**). Yield: 0.140 g (90%); white solid; m.p.: 53–55 °C (lit.: [48] 53–54 °C); ^1^H-NMR (200 MHz, CDCl_3_, ppm) δ = 7.89 (dd, *J* = 8.1, 1.4 Hz, 2H, H-Ar), 7.44–7.25 (m, 3H, H-Ar), 7.17 (ddd, *J* = 8.2, 7.3, 1.6 Hz, 1H, H-Ar), 6.94 (dd, *J* = 7.6, 1.6 Hz, 1H, H-Ar), 6.80–6.69 (m, 2H, H-Ar), 3.86 (s, 3H, -OCH_3_); ^13^C-NMR (50 MHz, CDCl_3_, ppm) δ = 158.2, 141.3, 133.7, 129.7, 128.7, 128.2, 122.5, 112.2, 109.8, 107.8, 56.0.

*Naphthalen-2-yl(phenyl)tellane* (**3f**). Yield: 0.146 g (88%); yellow oil; [55] ^1^H-NMR (200 MHz, CDCl_3_, ppm) δ = 8.19–8.14 (m, 1H, H-Ar), 7.97 (dd, *J* = 7.1, 1.1 Hz, 1H, H-Ar), 7.82–7.74 (m, 2H, H-Ar), 7.62–7.57 (m, 2H, H-Ar), 7.51–7.44 (m, 2H, H-Ar), 7.28–7.08 (m, 4H, H-Ar); ^13^C-NMR (101 MHz, CDCl_3_, ppm) δ = 138.9, 137.5, 135.9, 133.8, 131.8, 129.6, 128.9, 127.8, 127.1, 126.6, 126.4, 117.9, 114.9.

*1-(4-((4-Methoxyphenyl)tellanyl)phenyl)ethan-1-one* (**3g**). Yield: 0.152 g (86%); yellow oil; [57] ^1^H-NMR (200 MHz, CDCl_3_, ppm) δ = 7.78 (d, *J* = 8.8 Hz, 2H, H-Ar), 7.68 (d, *J* = 8.5 Hz, 2H, H-Ar), 7.48 (d, *J* = 8.5 Hz, 2H, H-Ar), 6.83 (d, *J* = 8.8 Hz, 2H, H-Ar), 3.82 (s, 3H, -OCH_3_), 2.52 (s, 3H, -COCH_3_); ^13^C-NMR (50 MHz, CDCl_3_, ppm) δ = 197.5, 160.4, 142.2, 135.5, 134.5, 128.6, 125.5, 115.8, 102.1, 55.2, 26.4.

*(3-Nitrophenyl)(p-tolyl)tellane* (**3h**). Yield: 0.142 g (83%); yellow oil; [57] ^1^H-NMR (200 MHz, CDCl_3_, ppm) δ = 8.41–8.29 (m, 1H, H-Ar), 8.06–8.01 (m, 1H, H-Ar), 7.82–7.72 (m, 3H, H-Ar), 7.30 (td, *J* = 8.3, 2.2 Hz, 1H, H-Ar), 7.12 (d, *J* = 7.1 Hz, 2H, H-Ar), 2.38 (s, 3H, -CH_3_); ^13^C-NMR (50 MHz, CDCl_3_, ppm) δ = 148.5, 141.8, 140.1, 139.5, 131.0, 130.4, 129.8, 122.2, 117.6, 109.1, 21.4.

*1-(4-((4-Chlorophenyl)tellanyl)phenyl)ethanone* (**3i**). Yield: 0.156 g (87%); yellow solid; m.p.: 67–68 °C (lit.: [57] 67–70 °C); ^1^H-NMR (200 MHz, CDCl_3_, ppm) δ = 7.76–7.72 (m, 4H, H-Ar), 7.61 (d, *J* = 7.9 Hz, 2H, H-Ar), 7.24 (d, *J* = 8.4 Hz, 2H, H-Ar), 2.56 (s, 3H, -COCH_3_); ^13^C-NMR (50 MHz, CDCl_3_, ppm) δ = 197.6, 140.9, 139.5, 136.3, 136.3, 136.2, 135.5, 130.3, 129.0, 123.5, 111.1, 26.6.

*3-((4-Chlorophenyl)tellanyl)aniline* (**3j**). Yield: 0.137 g (83%); brown solid; m.p.: 60–61 °C (lit.: [57] 58–60 °C); ^1^H-NMR (200 MHz, CDCl_3_, ppm) δ = 7.58 (d, *J* = 8.2 Hz, 2H, H-Ar), 7.18–6.95 (m, 5H, H-Ar), 6.59 (dt, *J* = 7.5, 1.9 Hz, 1H, H-Ar), 3.62 (s, 2H, -NH_2_); ^13^C-NMR (50 MHz, CDCl_3_, ppm) δ = 147.4, 139.2, 134. 2, 130.4, 129.7, 128.2, 124.4, 115.0, 112.6.

*1-(4-(Butyltellanyl)phenyl)ethan-1-one* (**3k**). Yield: 0.127 g (87%); yellow oil; [69] ^1^H-NMR (200 MHz, CDCl_3_, ppm) δ = 7.67 (d, *J* = 8.8 Hz, 2H, H-Ar), 6.76 (d, *J* = 8.8 Hz, 2H, H-Ar), 3.79 (s, 3H, -OCH_3_), 2.86–2.78 (m, 2H, -CH_2_-), 1.73 (p, *J* = 7.9, 7.4 Hz, 2H, -CH_2_-), 1.46–1.287 (m, 2H, -CH_2_-), 0.92–0.851 (m, 3H, -CH_3_); ^13^C-NMR (101 MHz, CDCl_3_, ppm) δ = 159.7, 141.0, 115.2, 100.7, 55.2, 34.0, 25.1, 13.5, 8.9.

*(4-Methoxyphenyl)(phenyl)selane* (**6a**). Yield: 0.114 g (87%); yellow oil; [54] ^1^H-NMR (200 MHz, CDCl_3_, ppm) δ = 7.51 (d, *J* = 8.9 Hz, 2H, H-Ar), 7.35–7.18 (m, 5H, H-Ar), 6.86 (d, *J* = 8.8 Hz, 2H, H-Ar), 3.81 (s, 3H, -OCH_3_); ^13^C-NMR (50 MHz, CDCl_3_, ppm) δ = 159.7, 136.5, 133.1, 130.9, 129.1, 126.4, 119.9, 115.1, 55.3.

*(4-Chlorophenyl)(phenyl)selane* (**6b**). Yield: 0.114 g (85%); transparent oil; [68] ^1^H-NMR (200 MHz, CDCl_3_, ppm) δ = 7.47–7.44 (m, 2H, H-Ar), 7.35 (d, *J* = 8.3 Hz, 2H, H-Ar), 7.27–7.18 (m, 5H, H-Ar); ^13^C-NMR (50 MHz, CDCl_3_, ppm) δ = 134.2, 133.6, 133.3, 130.8, 129.7, 129.6, 127.7.

*Phenyl(o-tolyl)selane* (**6c**). Yield: 0.110 g (89%); transparent oil; [70] ^1^H-NMR (200 MHz, CDCl_3_, ppm) δ = 7.42–7.36 (m, 2H, H-Ar), 7.34–7.14 (m, 6H, H-Ar), 7.09–7.01 (m, 1H, H-Ar), 2.40 (s, 3H, -CH_3_); ^13^C-NMR (50 MHz, CDCl_3_, ppm) δ = 139.9, 133.8, 132.8, 131.8, 130.9, 130.3, 129.4, 127.8, 127.2, 126.8, 22.4.

*Naphthalen-2-yl(phenyl)selane* (**6d**). Yield: 0.125 g (88%); yellow solid; m.p.: 68–70°C (lit.: [57] 69–71 °C); ^1^H-NMR (200 MHz, CDCl_3_, ppm) δ = 8.35–8.30 (m, 1H, H-Ar), 7.84–7.74 (m, 3H, H-Ar), 7.51–7.15 (m, 8H, H-Ar); ^13^C-NMR (50 MHz, CDCl_3_, ppm) δ = 134.2, 133.9, 131.8, 129.4, 129.3, 128.7, 127.8, 127.1, 126.9, 126.5, 126.1.

*(4-Chlorophenyl)(4-methoxyphenyl)selane* (**6e**). Yield: 0.132 g (89%); yellow solid; m.p.: 56–57 °C (lit.: [48] 58–59 °C); ^1^H-NMR (200 MHz, CDCl_3_, ppm) δ = 7.41 (d, *J* = 8.8 Hz, 2H, H-Ar), 7.18–7.06 (m, 4H, H-Ar), 6.77 (d, *J* = 8.8 Hz, 2H, H-Ar), 3.72 (s, 3H, -OCH_3_); ^13^C-NMR (50 MHz, CDCl_3_, ppm) δ = 160.0, 136.6, 132.5, 132.1, 131.6, 129.2, 119.5, 115.2, 55.3.

*(4-Chlorophenyl)(o-tolyl)selane* (**6f**). Yield: 0.117 g (83%); transparent oil; [57] ^1^H-NMR (200 MHz, CDCl_3_, ppm) δ = 7.36–7.03 (m, 8H, H-Ar), 2.37 (s, 3H, -CH_3_); ^13^C-NMR (50 MHz, CDCl_3_, ppm) δ = 140.2, 134.1, 133.8, 133.4, 131.3, 130.5, 129.6, 129.4, 128.3, 127.0, 22.5.

*(4-Chlorophenyl)(o-tolyl)selane* (**6g**). Yield: 0.130 g (89%); transparent oil; [57] ^1^H-NMR (200 MHz, CDCl_3_, ppm) δ = 7.55 (d, *J* = 8.8 Hz, 2H, H-Ar), 7.16–7.05 (m, 1H, H-Ar), 6.88 (d, *J* = 8.8 Hz, 2H, H-Ar), 6.82–6.72 (m, 3H, H-Ar), 3.88 (s, 3H, -OCH_3_), 3.81 (s, 3H, -OCH_3_); ^13^C-NMR (50 MHz, CDCl_3_, ppm) δ = 160.3, 156.0, 138.5, 129.1, 127.0, 123.6, 121.7, 117.4, 115.4, 110.2, 55.9, 55.4.

*Butyl(4-methoxyphenyl)selane (4-chlorophenyl)(o-tolyl)selane* (**6h**). Yield: 0.094 g (77%); yellow oil; [48] ^1^H-NMR (200 MHz, CDCl_3_, ppm) δ = 7.46 (d, *J* = 8.9 Hz, 2H), 6.81 (d, *J* = 8.8 Hz, 2H), 3.79 (s, 3H, -OCH_3_), 2.82 (t, *J* = 7.6 Hz, 2H, -CH_2_-), 1.71–1.576 (m, 2H, -CH_2_-), 1.49–1.34 (m, 2H, -CH_2_-), 0.89 (t, *J* = 7.2 Hz, 3H, -CH_3_); ^13^C-NMR (50 MHz, CDCl_3_, ppm) δ = 159.2, 135.6, 120.4, 114.8, 55.4, 32.5, 29.0, 23.0, 13.7.

*(4-Methoxyphenyl)(phenyl)sulfane* (**7a**). Yield: 0.077 g (71%); transparent oil; [68] ^1^H-NMR (200 MHz, CDCl_3_, ppm) δ = 7.42 (d, *J* = 8.9 Hz, 2H, H-Ar), 7.28–7.15 (m, 5H, H-Ar), 6.90 (d, *J* = 8.9 Hz, 2H, H-Ar), 3.82 (s, 3H, -OCH_3_); ^13^C-NMR (50 MHz, CDCl_3_, ppm) δ = 160.0, 138.7, 135.5, 129.0, 128.3, 125.9, 124.5, 115.1, 55.5.

*(3-Chlorophenyl)(4-methoxyphenyl)sulfane* (**7b**). Yield: 0.085 g (68%); white solid; m.p.: 59–61 °C (lit.: [57] 59–61 °C); ^1^H-NMR (400 MHz, CDCl_3_, ppm) δ = 7.42 (d, *J* = 8.9 Hz, 2H, H-Ar), 7.13–7.03 (m, 3H, H-Ar), 6.98 (dt, *J* = 7.7, 1.3 Hz, 1H, H-Ar), 6.91 (d, *J* = 8.9 Hz, 2H, H-Ar), 3.81 (s, 3H, -OCH_3_); ^13^C-NMR (50 MHz, CDCl_3_, ppm) δ = 160.4, 141.4, 136.2, 134.9, 129.9, 127.2, 125.7, 125.6, 122.8, 115.4, 55.5.

## 4. Conclusions

In summary, we have developed a straightforward method for the preparation of unsymmetrical diorganyl chalcogenides (S, Se and Te), under solvent- and ligand-free conditions. Remarkably, copper iodide was found to be a highly efficient catalyst for this cross-coupling reaction, affording the desired products in very high yields in the absence of solvent under microwave irradiation. Of particular importance, this new metal-catalyzed process showed good potential applicability within a very broad scope, without the need for the use of ligands or solvents. It appears that the chemistry described herein characterizes a useful alternative approach to the synthesis of organochalcogen compounds through a copper-catalyzed processes.

## Figures and Tables

**Table 1:**
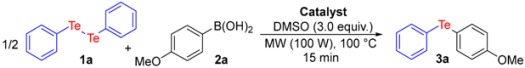
Optimization of reaction conditions ^a^.

Entry	Catalyst	Amount	Yield (%) ^b,c^
1	CuI	1.0 mol %	43
2	CuI	2.0 mol %	71
3	CuI	3.0 mol %	90
4	CuI	4.0 mol %	90
5	CuBr	3.0 mol %	56
6	CuCl	3.0 mol %	67
7	CuCl_2_	3.0 mol %	72
8	Cu(OAc)_2_	3.0 mol %	46
9	CuO	3.0 mol %	61
10	nano-CuO	3.0 mol %	65
11	I_2_	3.0 mol %	69
12	-	-	Traces

^a^ Reaction conditions: **1a** (0.25 mmol), **2a** (2.0 equiv.), DMSO (3.0 equiv.) under MW irradiation; ^b^ Isolated yield; ^c^ This reaction works similarly in 0.125 mmol scale with less reproducibility.

**Table 2:**
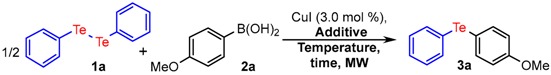
Optimization of reaction conditions ^a^.

Entry	Additive (equiv.)	MW (W)	T (°C)	Time (min)	Yield (%) ^b^
1	DMSO (3.0)	100	100	15	90
2	CH_3_CN (3.0)	100	100	15	35
3	EtOH (3.0)	100	100	15	25
4	Dioxane (3.0)	100	100	15	40
5	H_2_O (3.0)	100	100	15	-
6	TBHP (3.0)	100	100	15	-
7	-	100	100	15	10
8 ^c^	DMSO (3.0)	100	100	15	29
9	DMSO (2.0)	100	100	15	78
10	DMSO (4.0)	100	100	15	90
11	DMSO (3.0)	100	100	10	73
12	DMSO (3.0)	100	100	20	88
13	DMSO (3.0)	100	80	15	68
14	DMSO (3.0)	100	120	15	77
15	DMSO (3.0)	120	100	15	86
16	DMSO (3.0)	80	100	15	70
17 ^d^	DMSO (3.0)	-	100	24 h	57

^a^ Reaction conditions: **1a** (0.25 mmol), **2a** (2.0 equiv.), CuI (3.0 mol %), DMSO (3.0 equiv.) under MW irradiation; ^b^ Isolated yield; ^c^ Reaction under argon atmosphere; ^d^ Conventional heating.

## Supplementary Materials

Copies of the ^1^H- and ^13^C-NMR spectra are available online.
