# Supplementary material for: Copper-Catalyzed Synthesis of Unsymmetrical Diorganyl Chalcogenides (Te/Se/S) from Boronic Acids under Solvent-Free Conditions ‡
Source: Molecules. 2017 Aug 18;22(8):1367. doi: 10.3390/molecules22081367 (PMC6152410; doi:10.3390/molecules22081367)
Supplement: Supplementary file 1 [file molecules-22-01367-s001.pdf]

molecules

MDPI

Supplementary material

Copper-catalyzed synthesis of unsymmetrical diorganyl chalcogenides (Te/Se/S) from boronic acids under solvent-free conditions

Sumbal Saba [1,2], Giancarlo V. Botteselle [3], Marcelo Godoi [4], Tiago E. A. Frizon [5], Fábio Z. Galetto [1], Jamal Rafique [1,*] and Antonio L. Braga [1,*]

[1] Departamento de Química, Universidade Federal de Santa Catarina, UFSC, Florianopolis 88040-900, SC, Brazil
[2] Department of chemistry, Shaheed Benazir Bhutto Women University, Peshawar, 25000, KPK, Pakistan
[3] Laboratório de Pesquisa Química, CECE, Universidade Estadual do Oeste do Paraná UNIOESTE, 85819-110, PR, Brazil
[4] Escola de Química e Alimentos, Universidade Federal do Rio Grande-Campus Santo Antônio da Patrulha, RS, Brazil
[5] Universidade Federal de Santa Catarina, UFSC, Campus Araranguá, 88905-120, SC, Brazil

**E-mail*: jamal.chm@gmail.com (J.R.); braga.antonio@ufsc.br (A.L.B.)

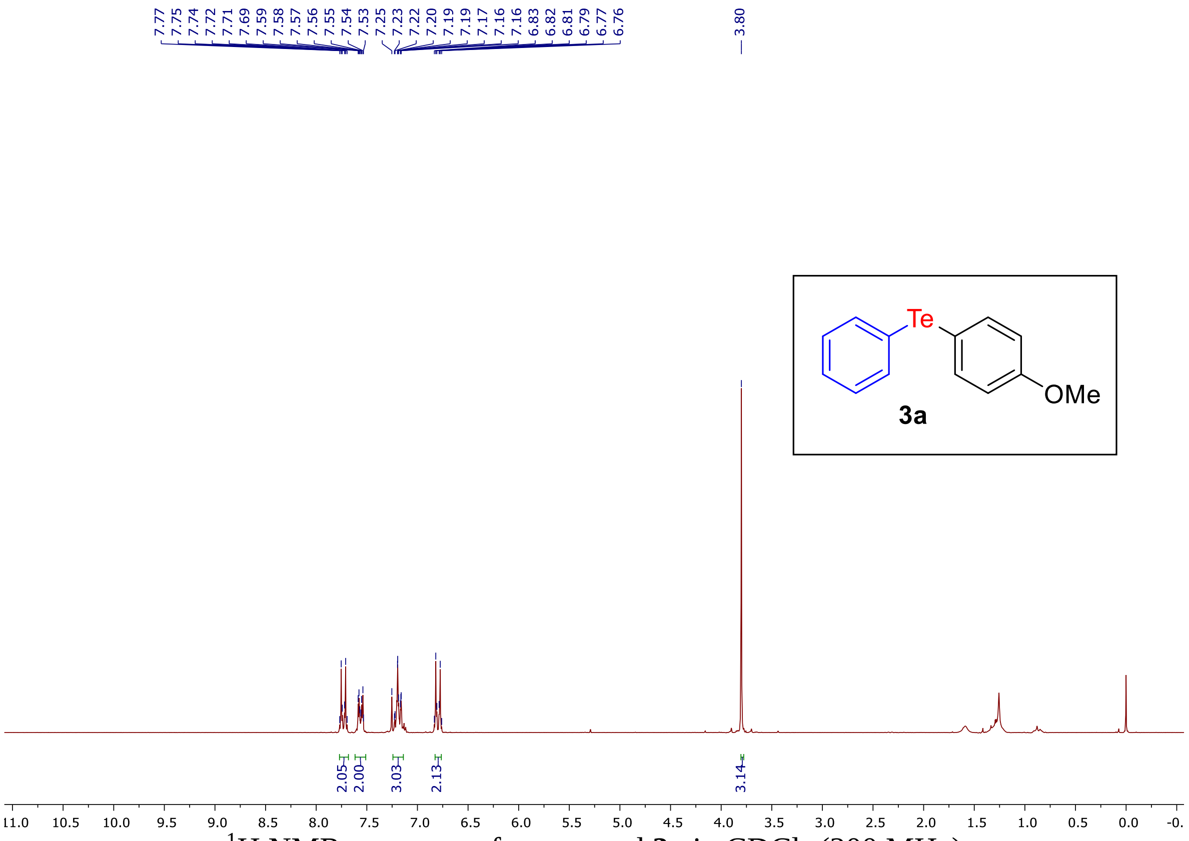

^{1}H NMR spectrum of compound **3a** in $CDCl_3$ (200 MHz)

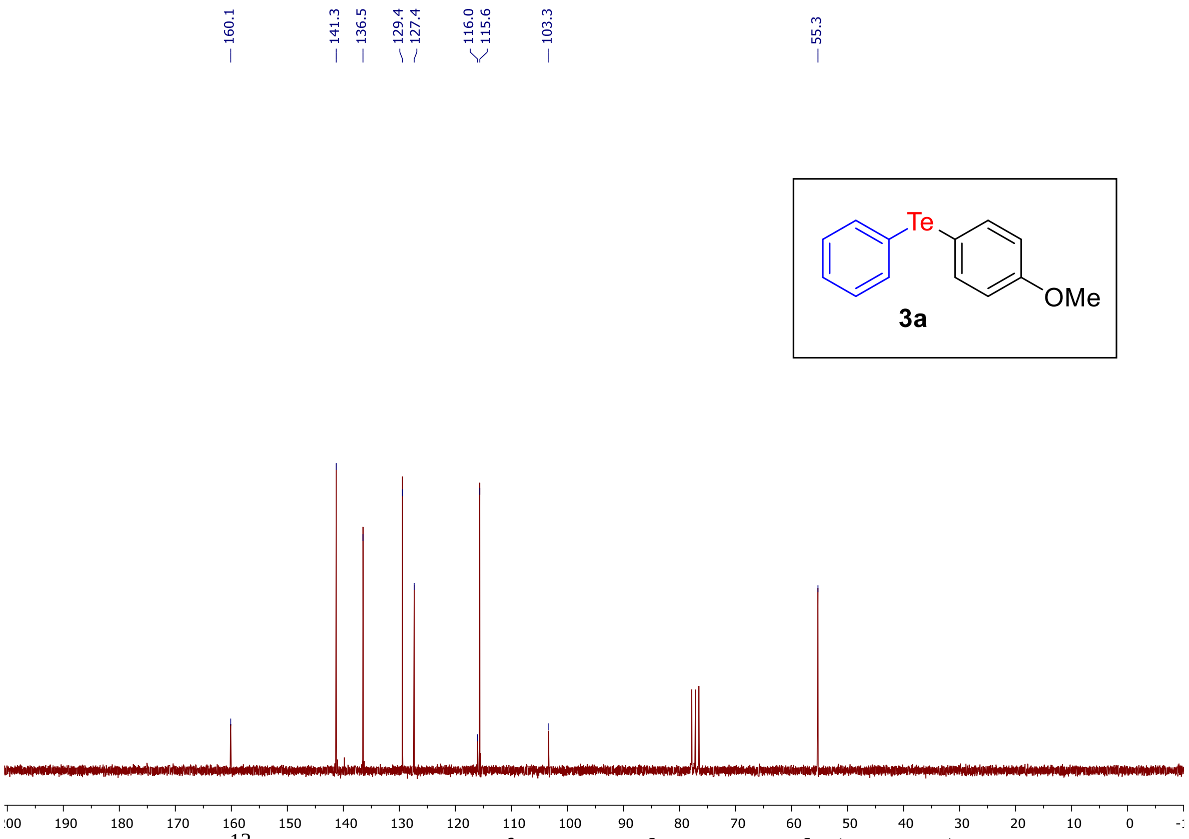

^{13}C NMR spectrum of compound **3a** in $CDCl_3$ (50 MHz)

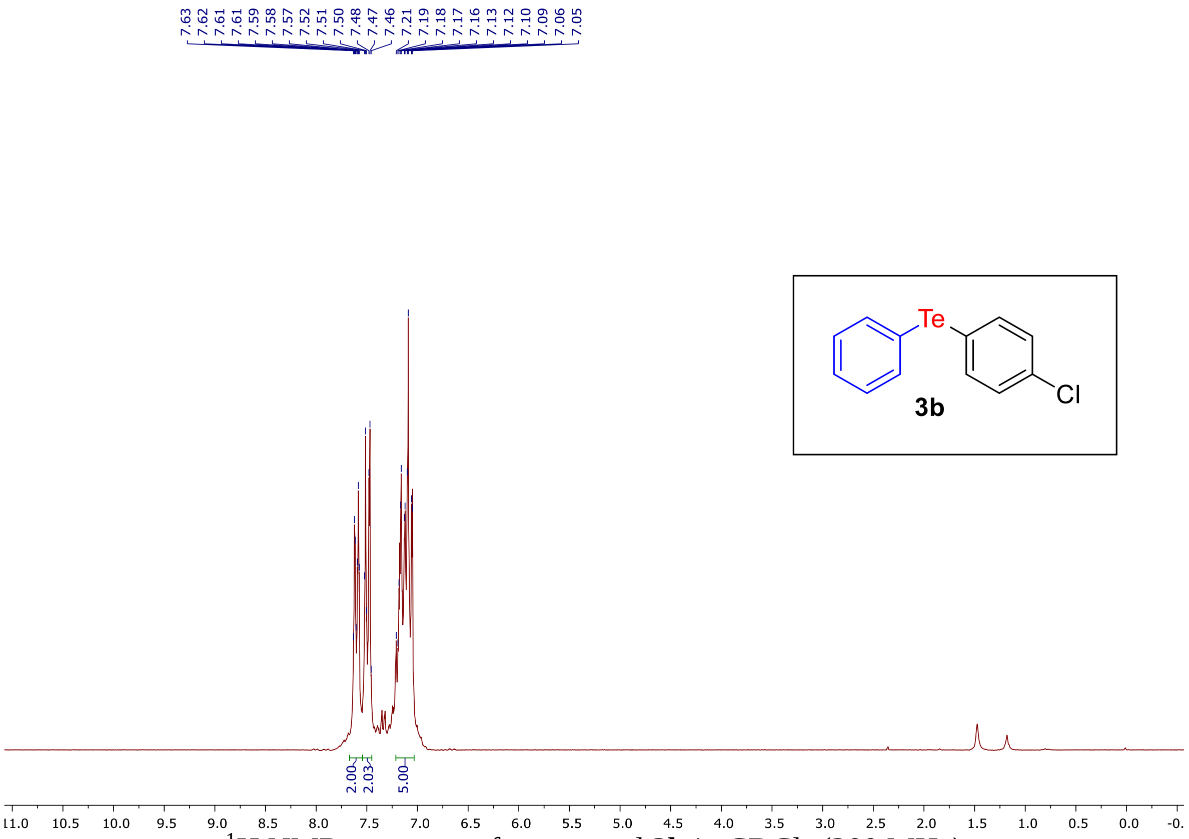

^{1}H NMR spectrum of compound **3b** in $CDCl_3$ (200 MHz)

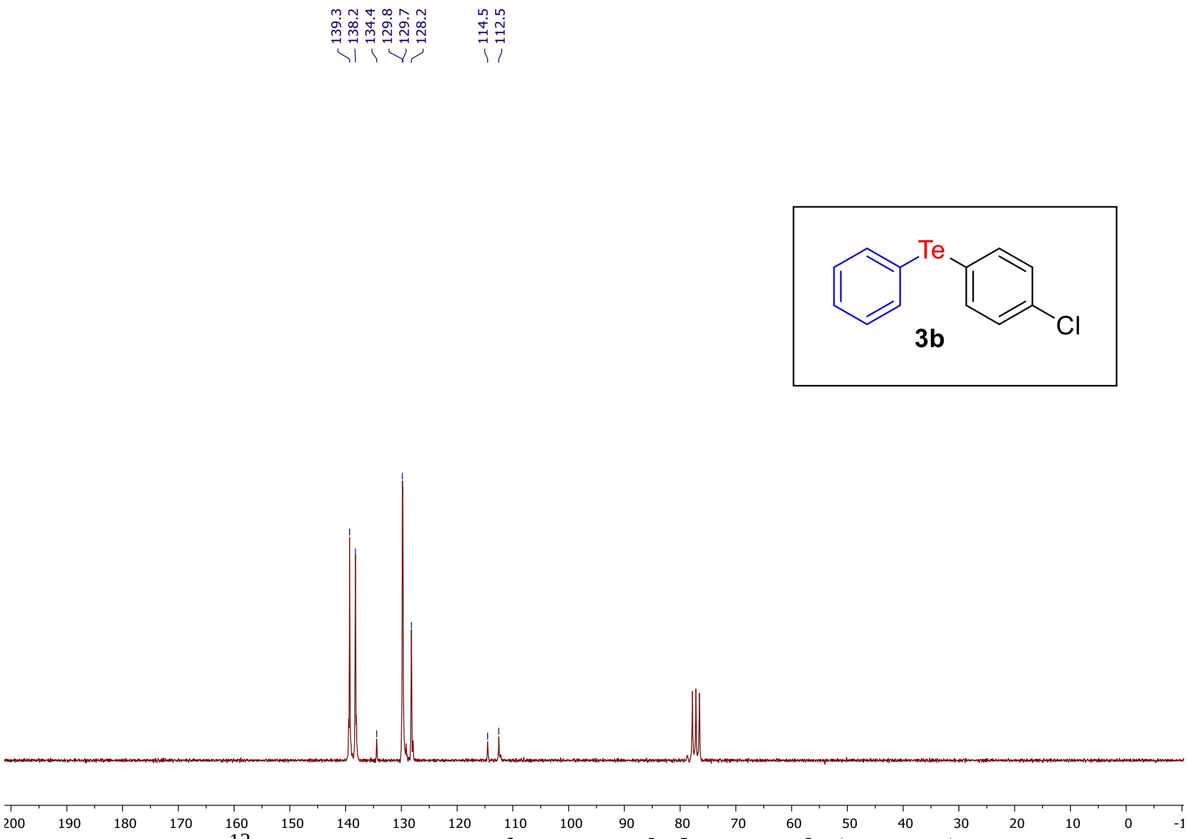

^{13}C NMR spectrum of compound **3b** in $CDCl_3$ (50 MHz)

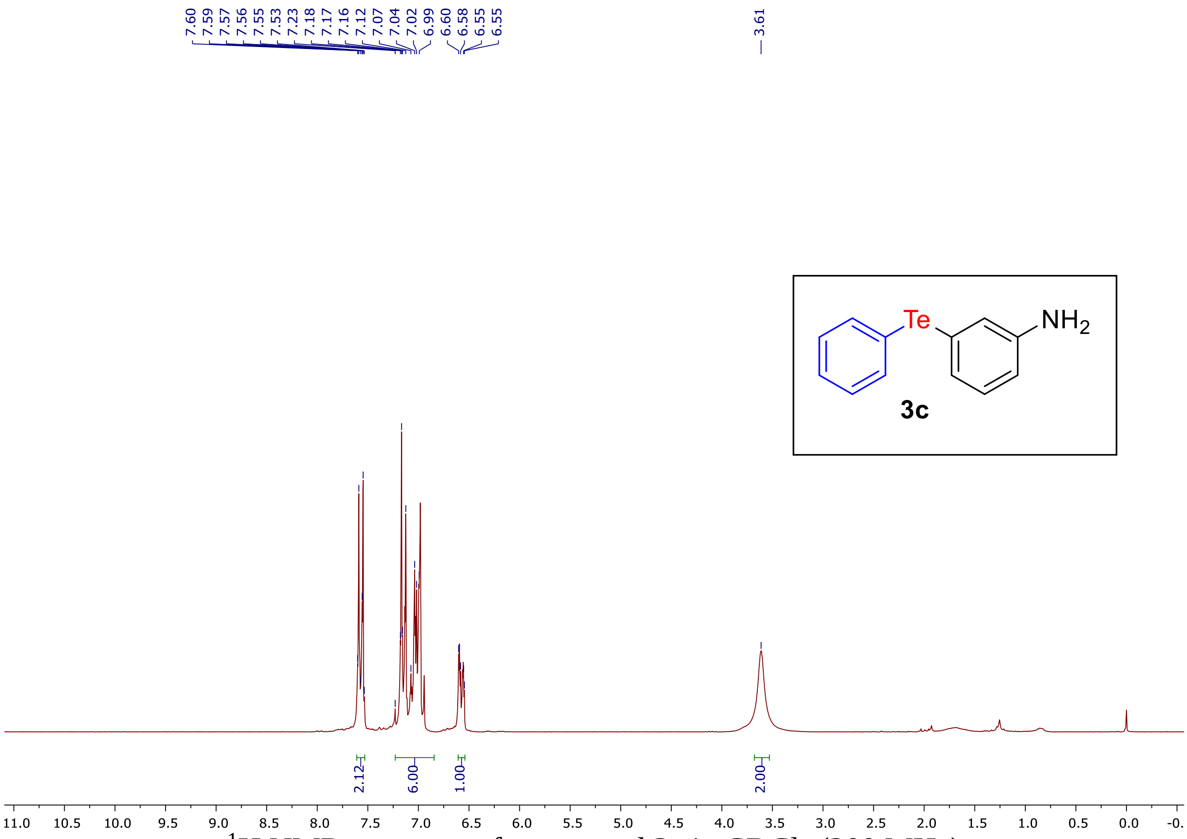

^{1}H NMR spectrum of compound **3c** in $CDCl_3$ (200 MHz)

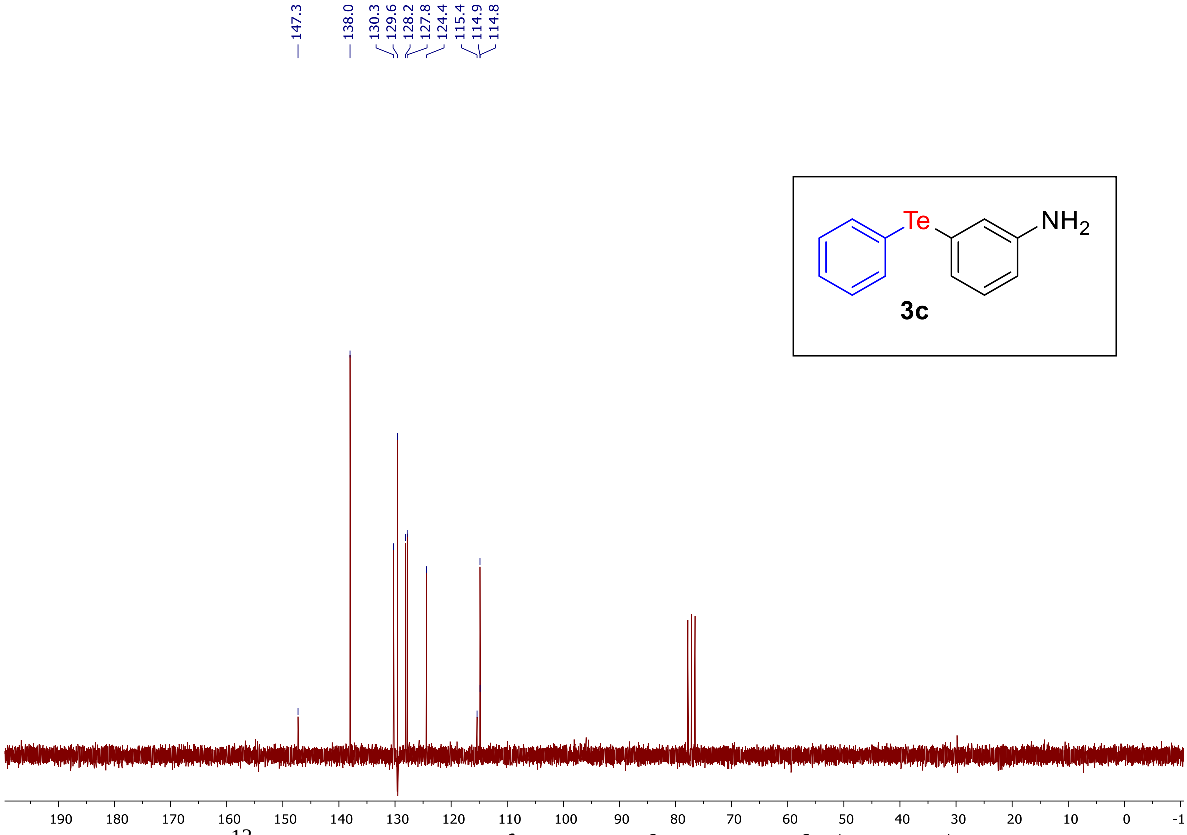

^{13}C NMR spectrum of compound **3c** in $CDCl_3$ (50 MHz)

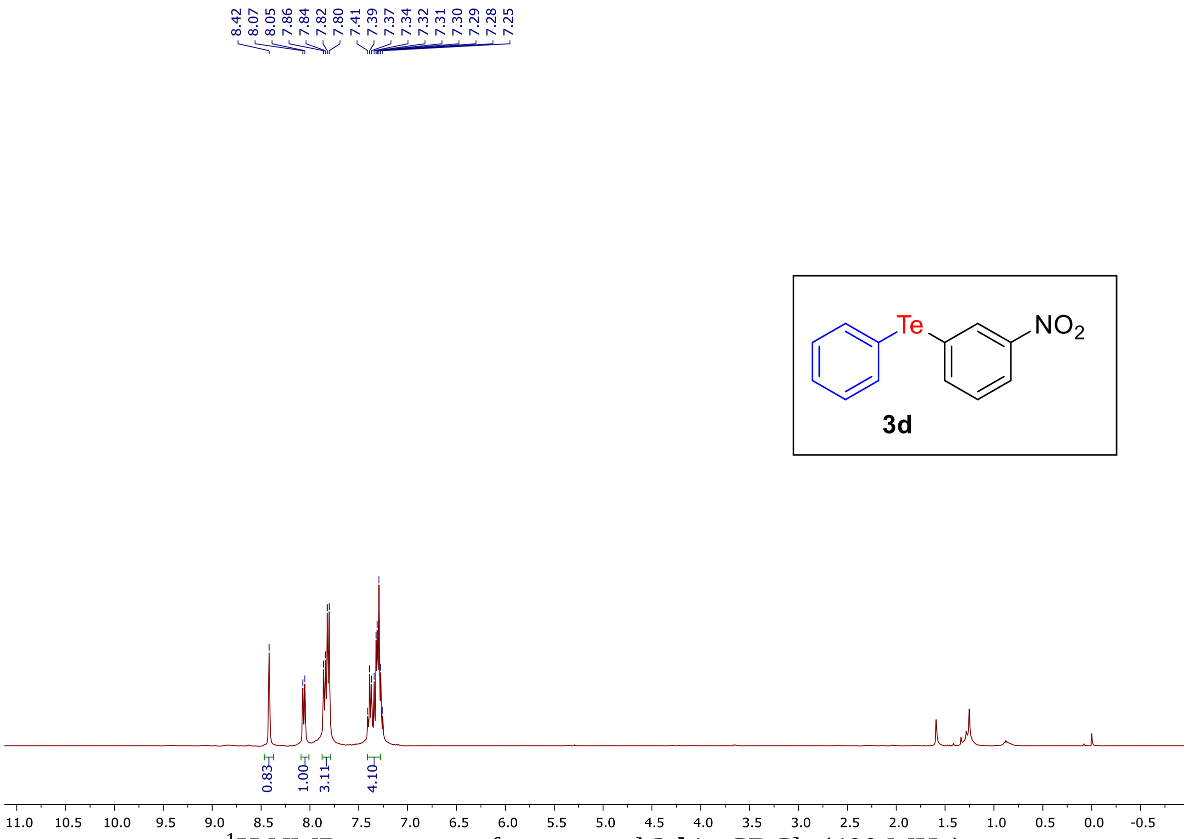

^{1}H NMR spectrum of compound **3d** in $CDCl_3$ (400 MHz)

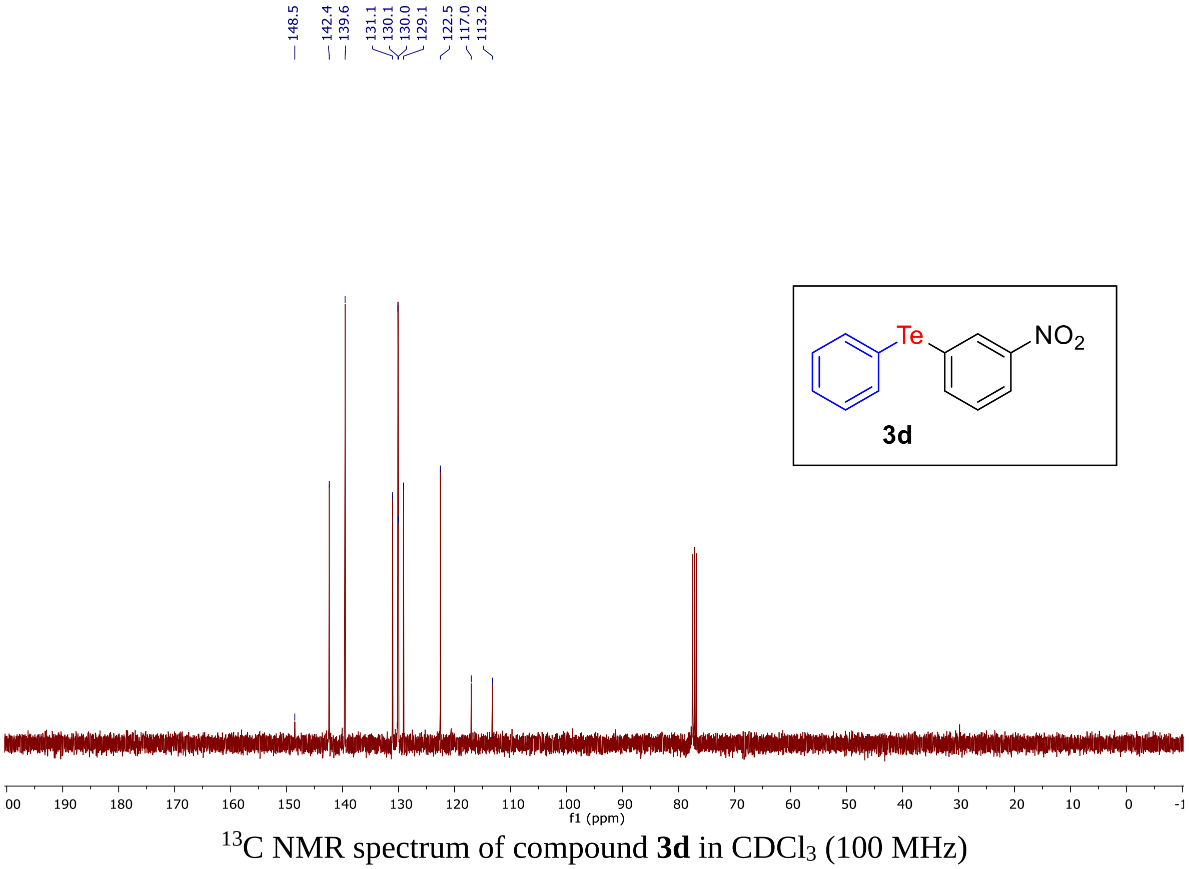

^{13}C NMR spectrum of compound **3d** in $CDCl_3$ (100 MHz)

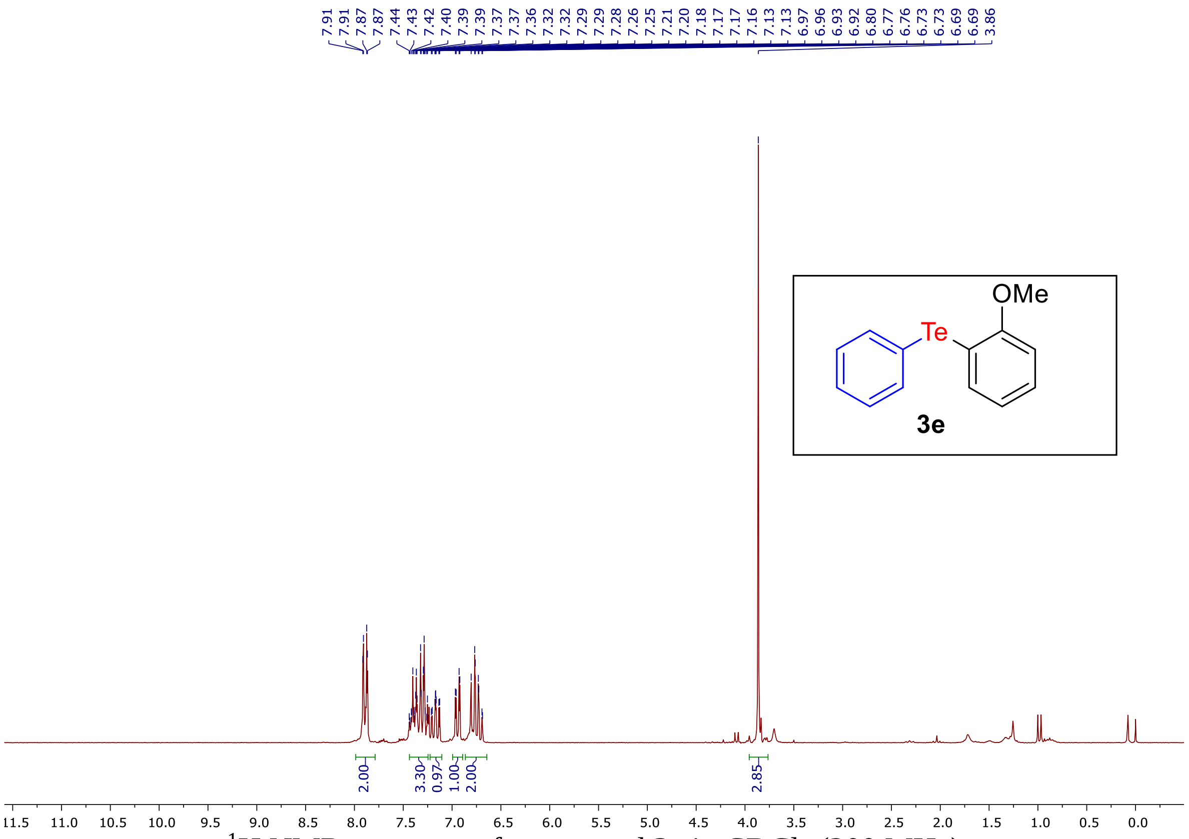

^{1}H NMR spectrum of compound **3e** in $CDCl_3$ (200 MHz)

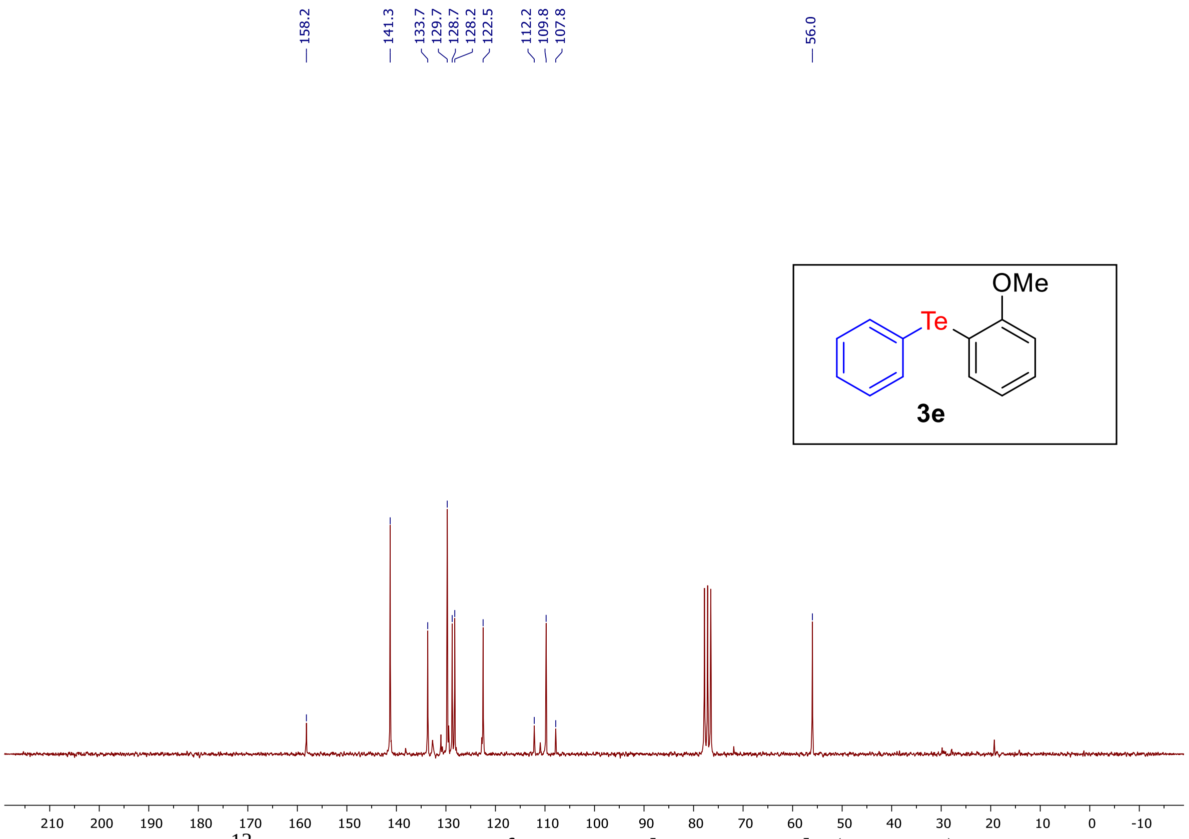

^{13}C NMR spectrum of compound **3e** in $CDCl_3$ (50 MHz)

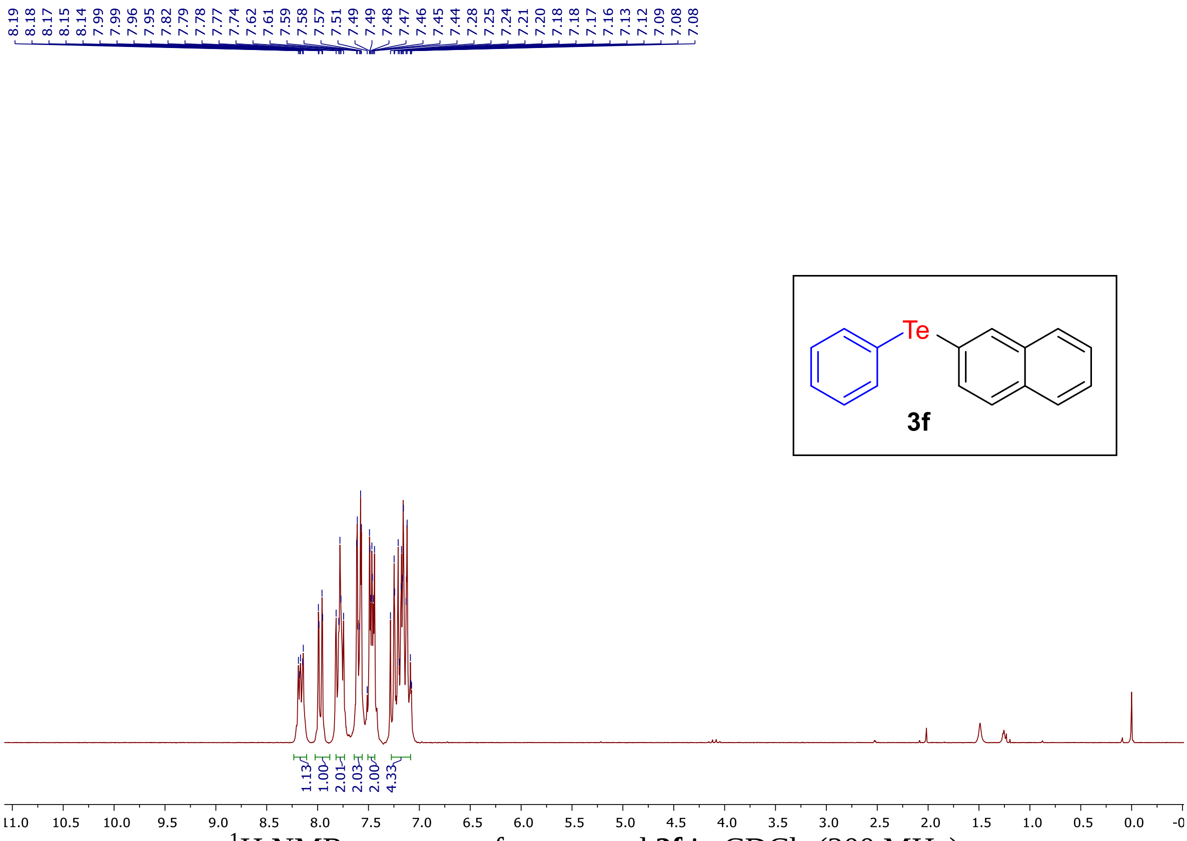

^{1}H NMR spectrum of compound **3f** in $CDCl_3$ (200 MHz)

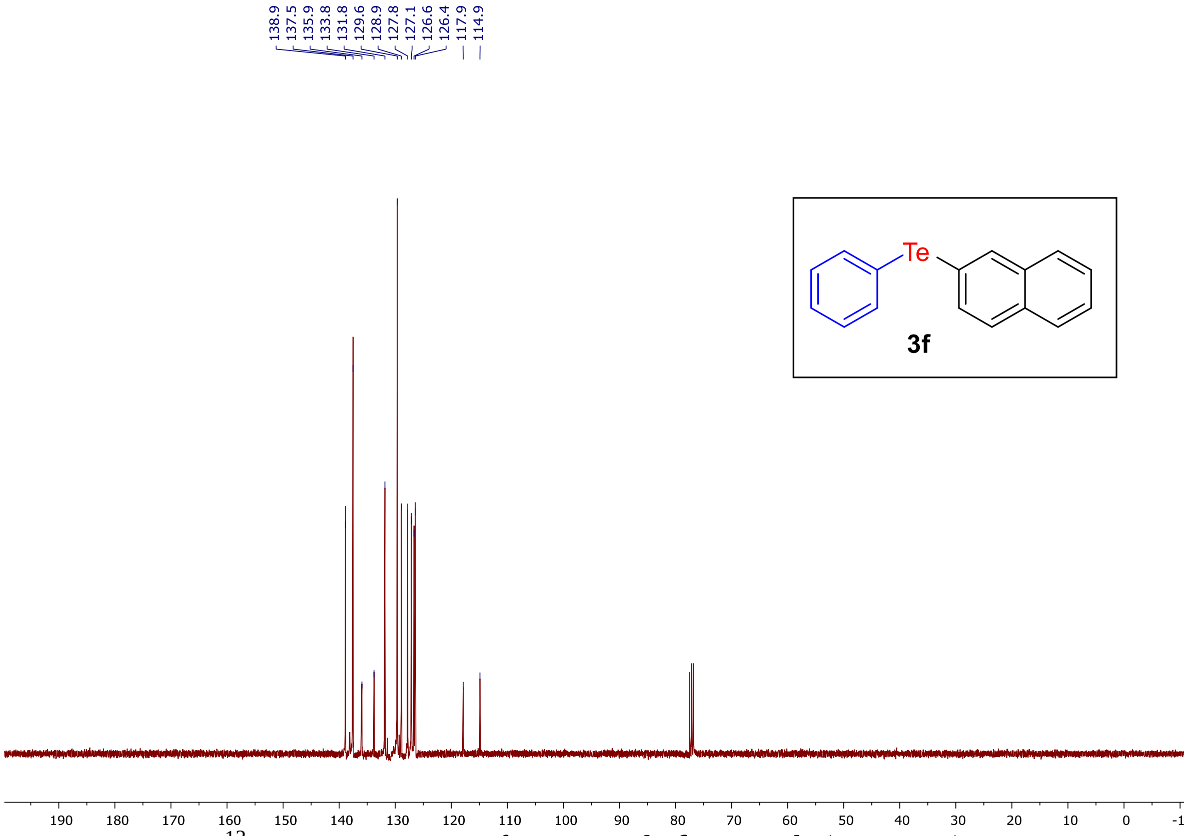

^{13}C NMR spectrum of compound **3f** in $CDCl_3$ (100 MHz)

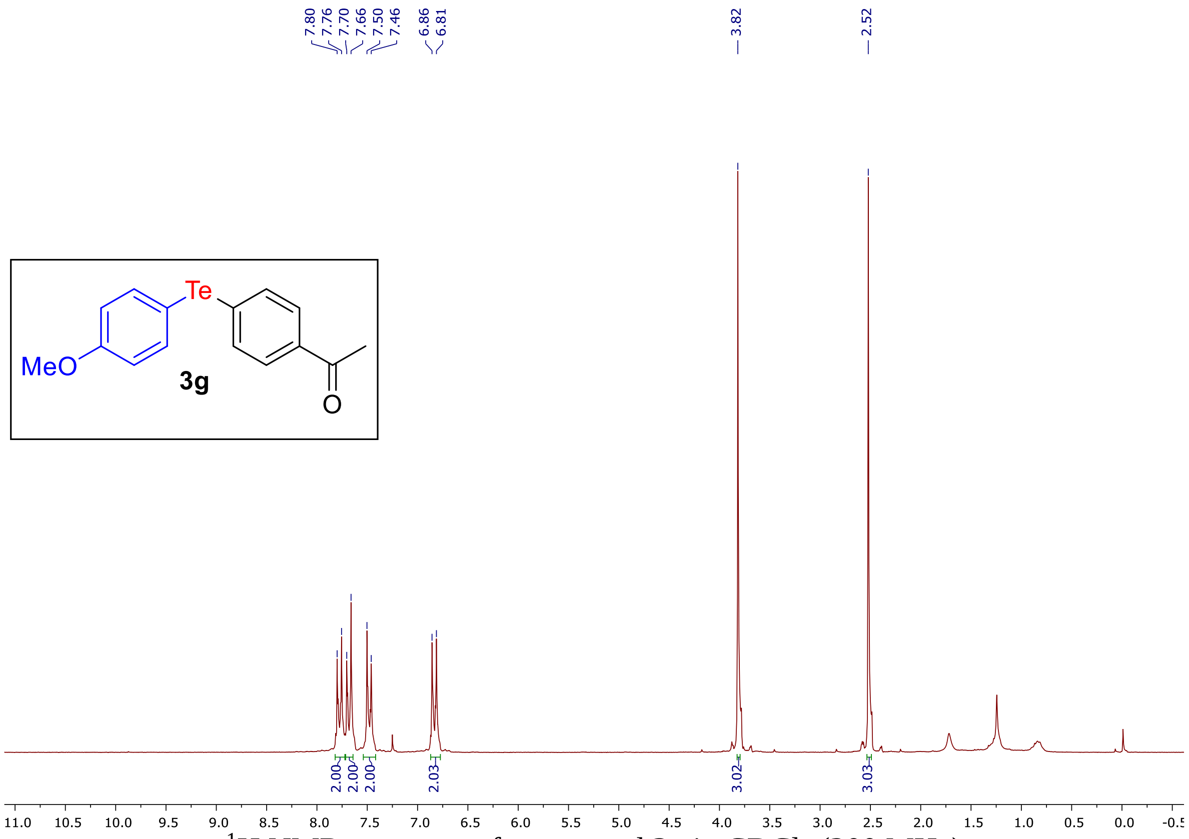

^{1}H NMR spectrum of compound **3g** in $CDCl_3$ (200 MHz)

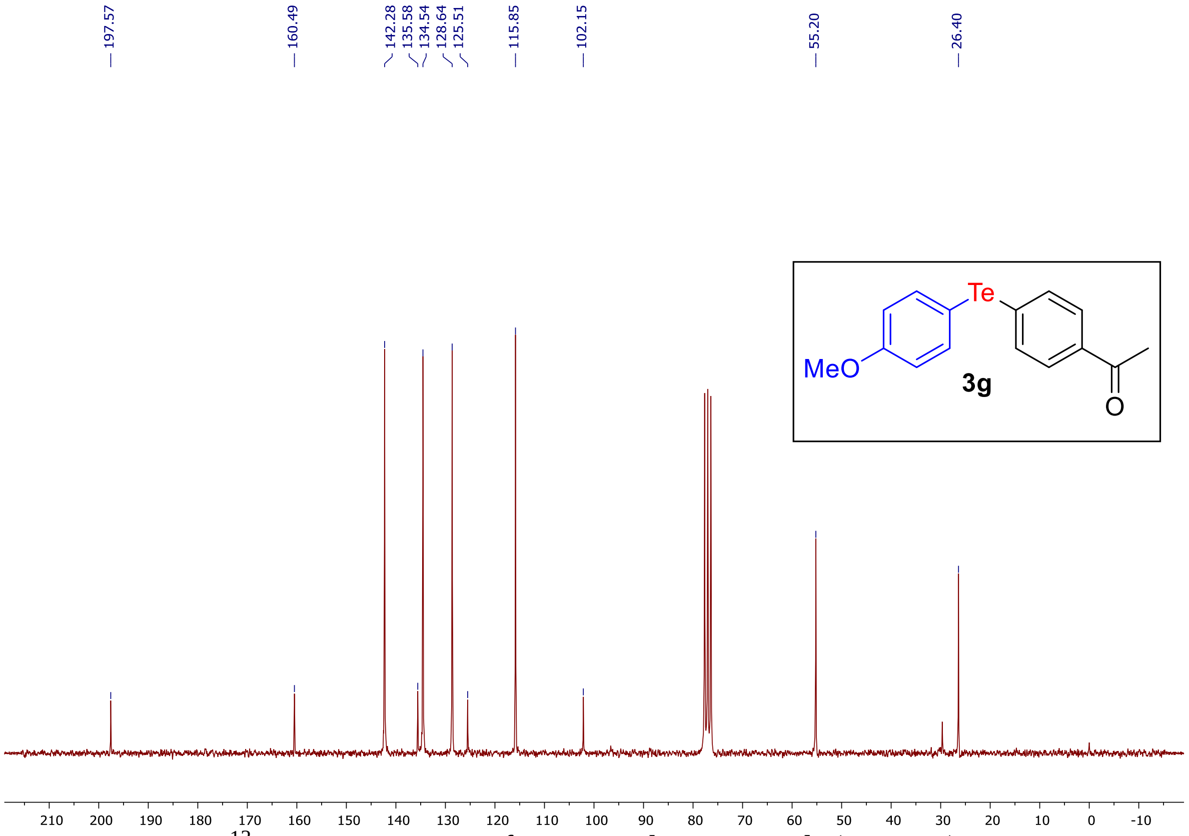

^{13}C NMR spectrum of compound **3g** in $CDCl_3$ (50 MHz)

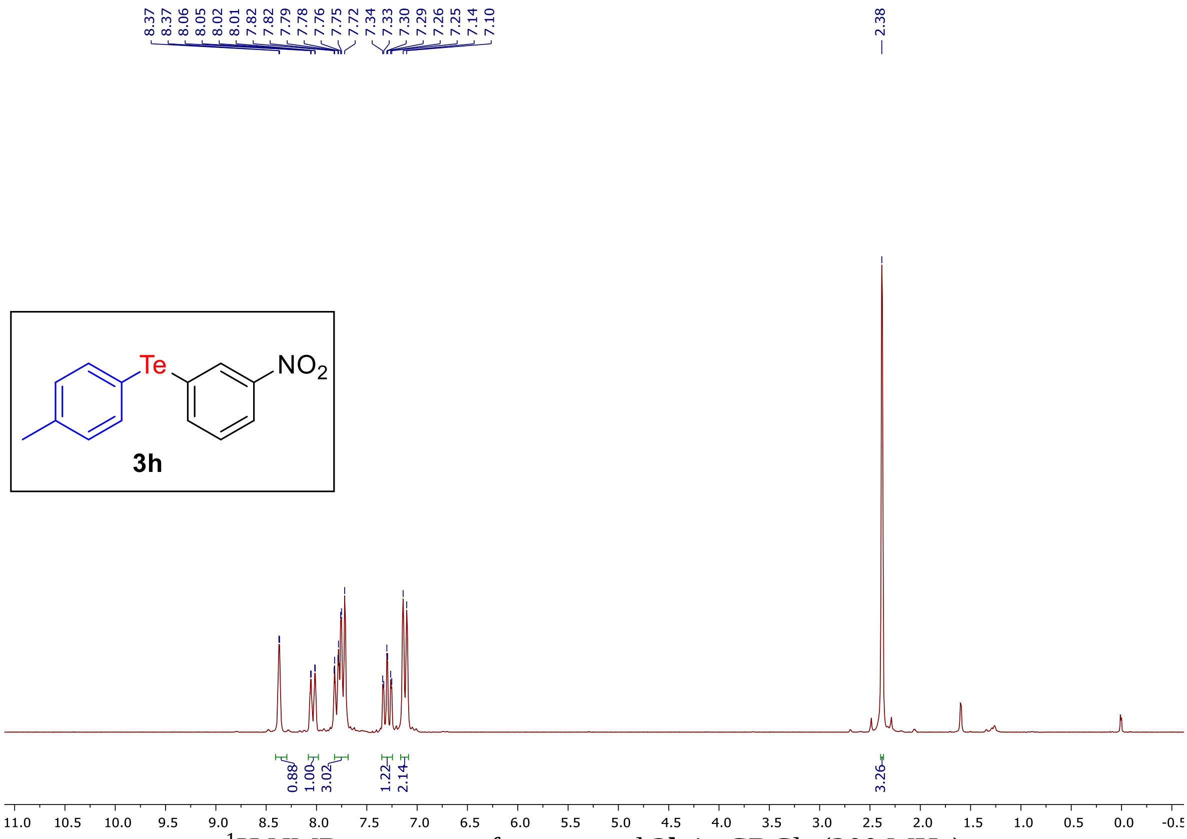

^{1}H NMR spectrum of compound **3h** in $CDCl_3$ (200 MHz)

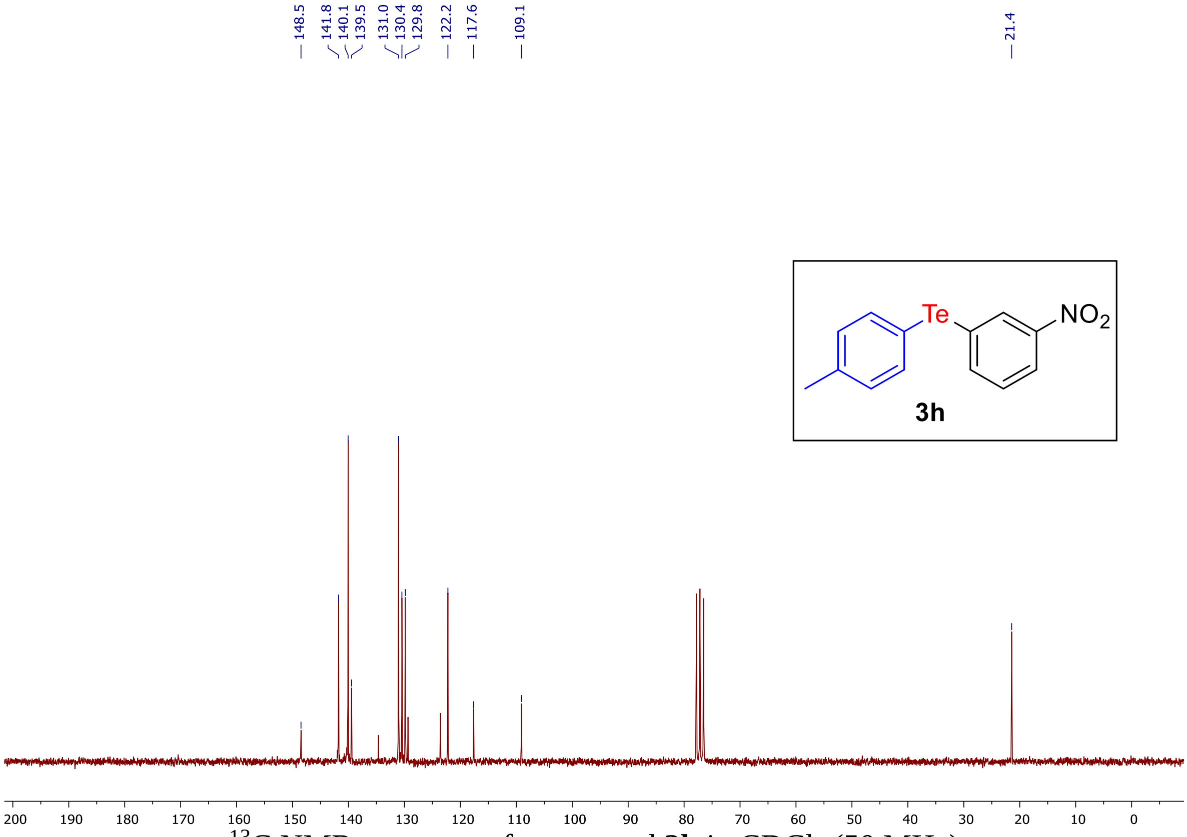

^{13}C NMR spectrum of compound **3h** in $CDCl_3$ (50 MHz)

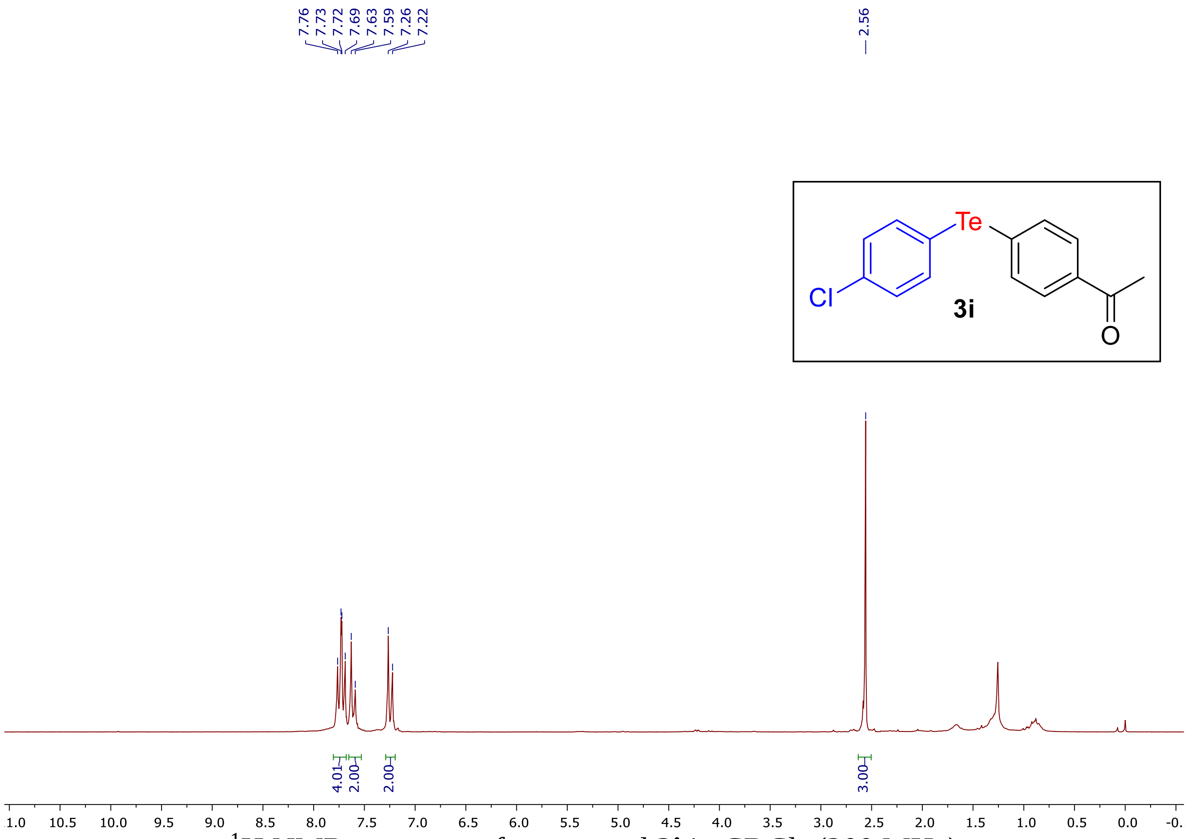

^{1}H NMR spectrum of compound **3i** in $CDCl_3$ (200 MHz)

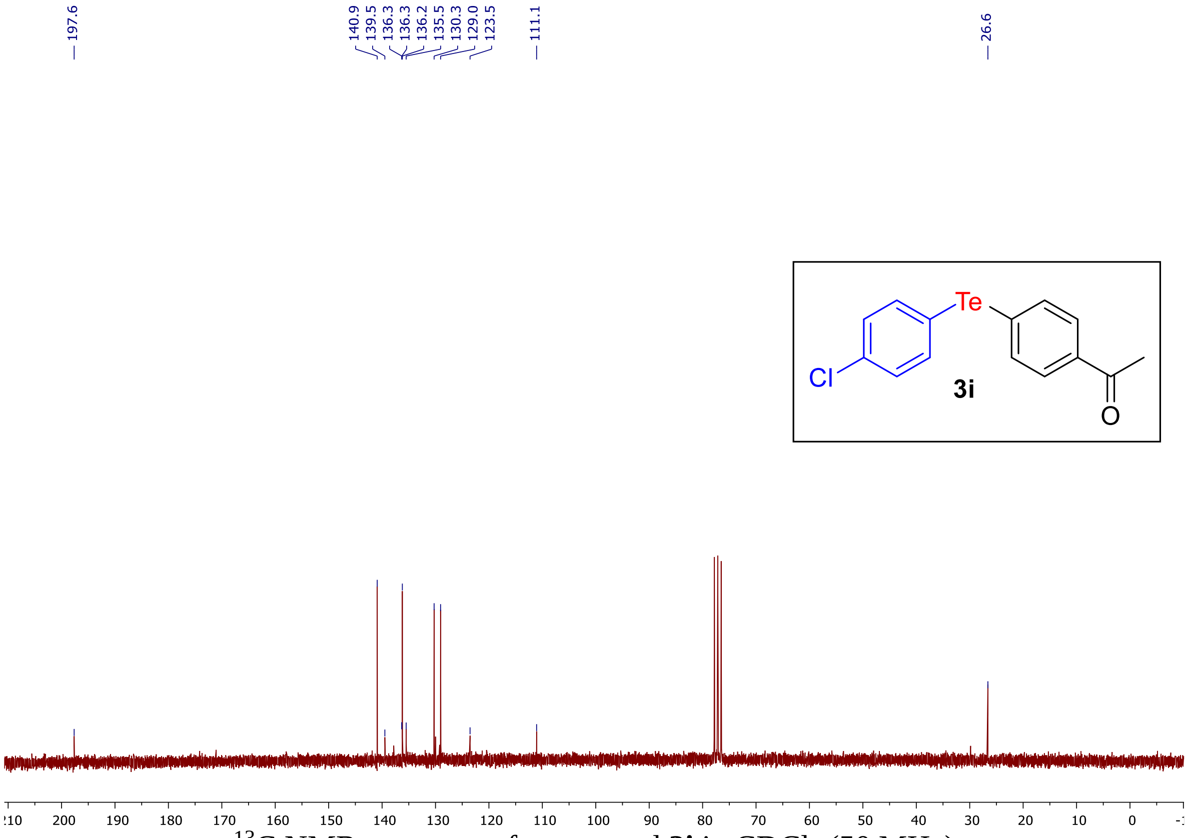

^{13}C NMR spectrum of compound **3i** in $CDCl_3$ (50 MHz)

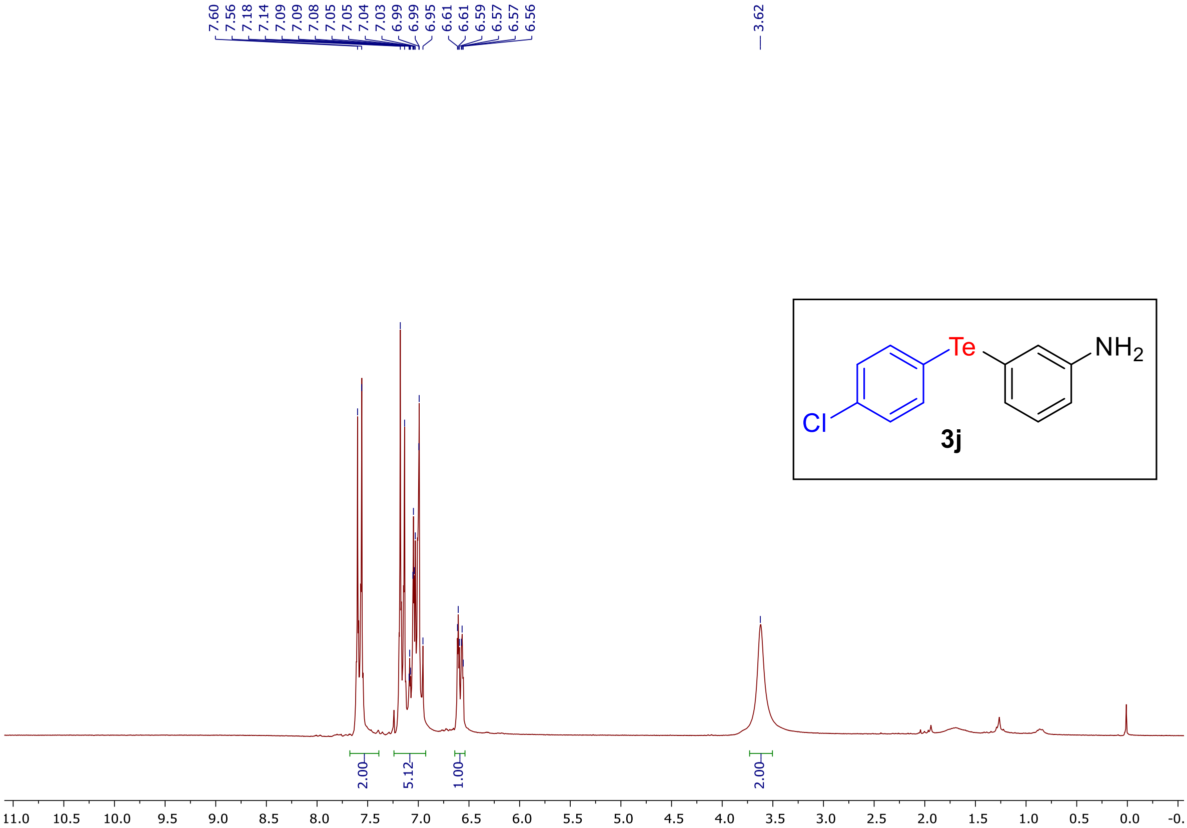

^{1}H NMR spectrum of compound **3j** in $CDCl_3$ (200 MHz)

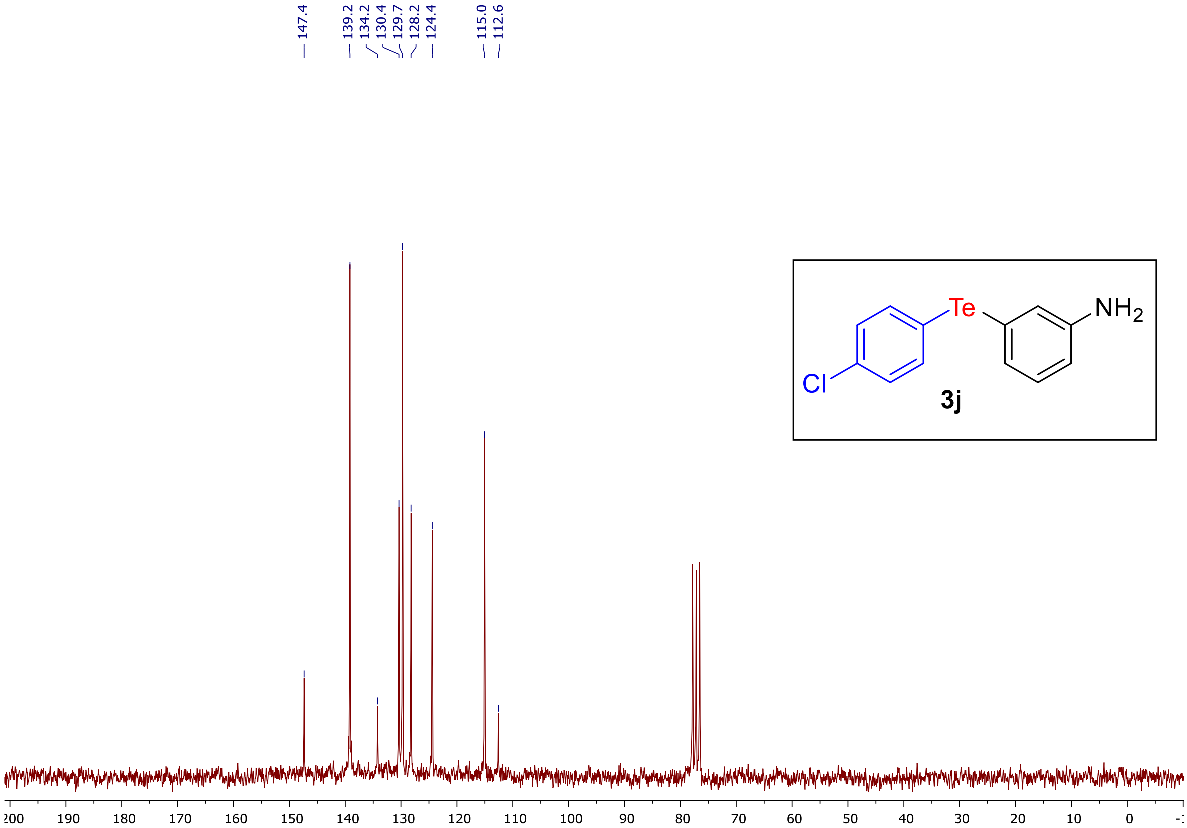

^{13}C NMR spectrum of compound **3j** in $CDCl_3$ (50 MHz)

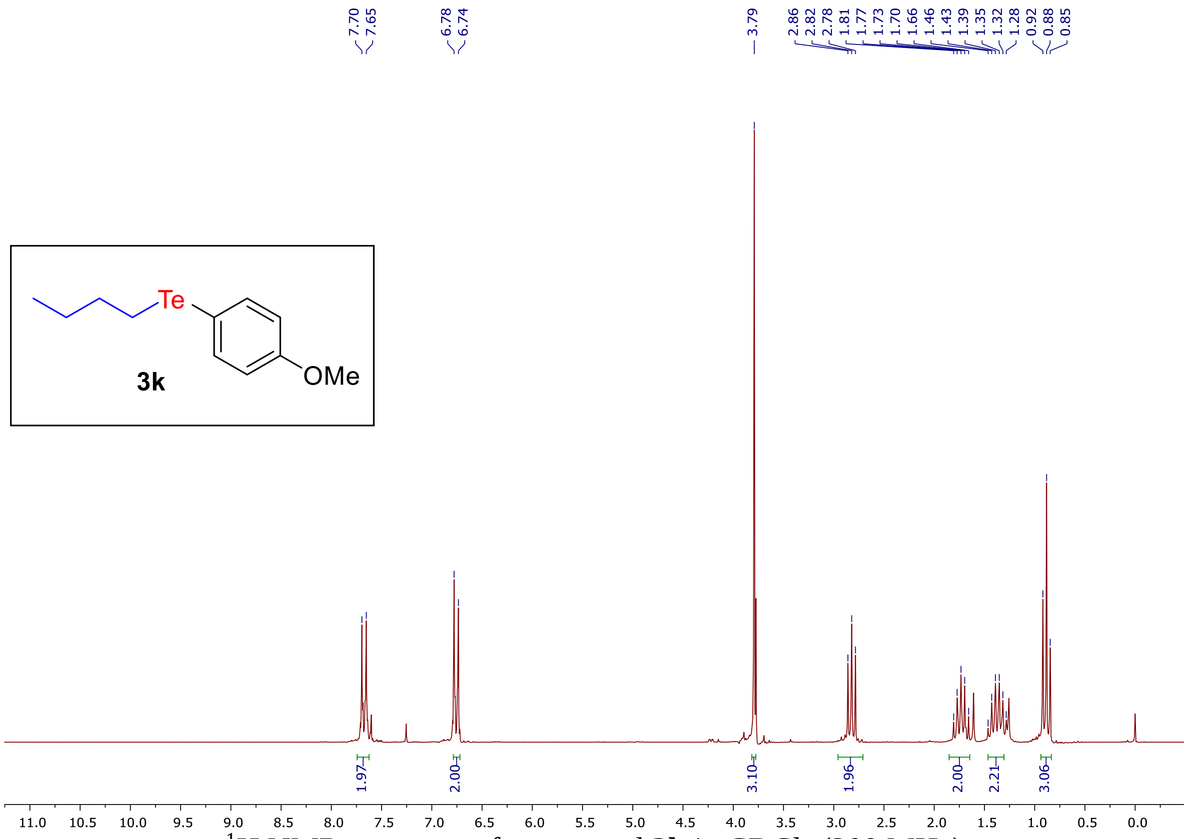

^{1}H NMR spectrum of compound **3k** in $CDCl_3$ (200 MHz)

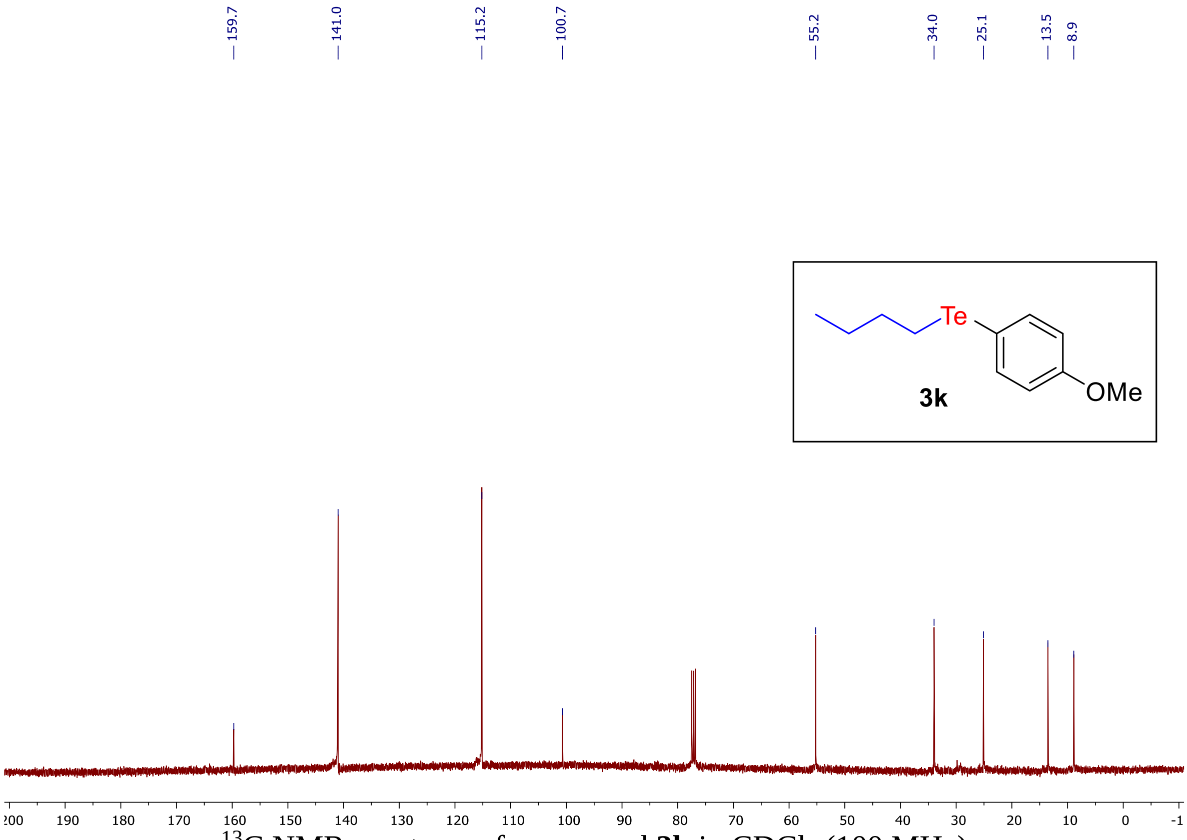

^{13}C NMR spectrum of compound **3k** in $CDCl_3$ (100 MHz)

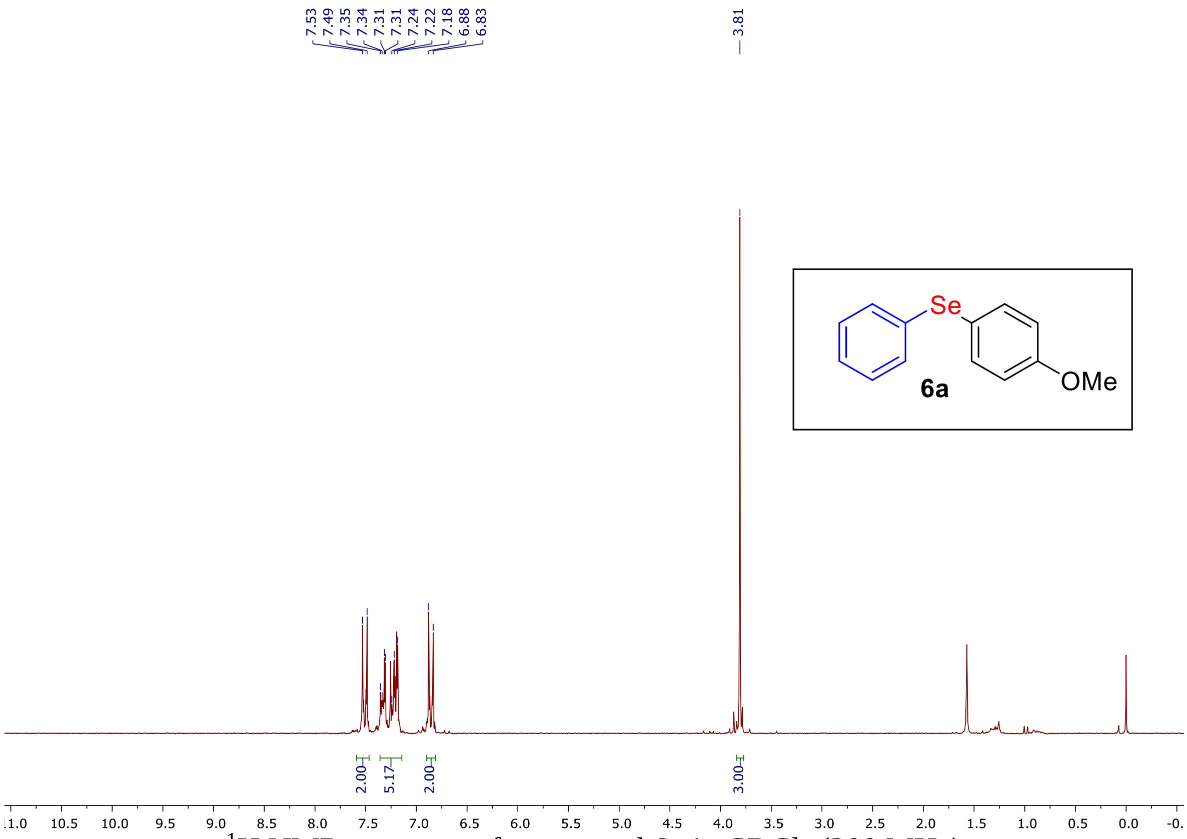

^{1}H NMR spectrum of compound **6a** in $CDCl_3$ (200 MHz)

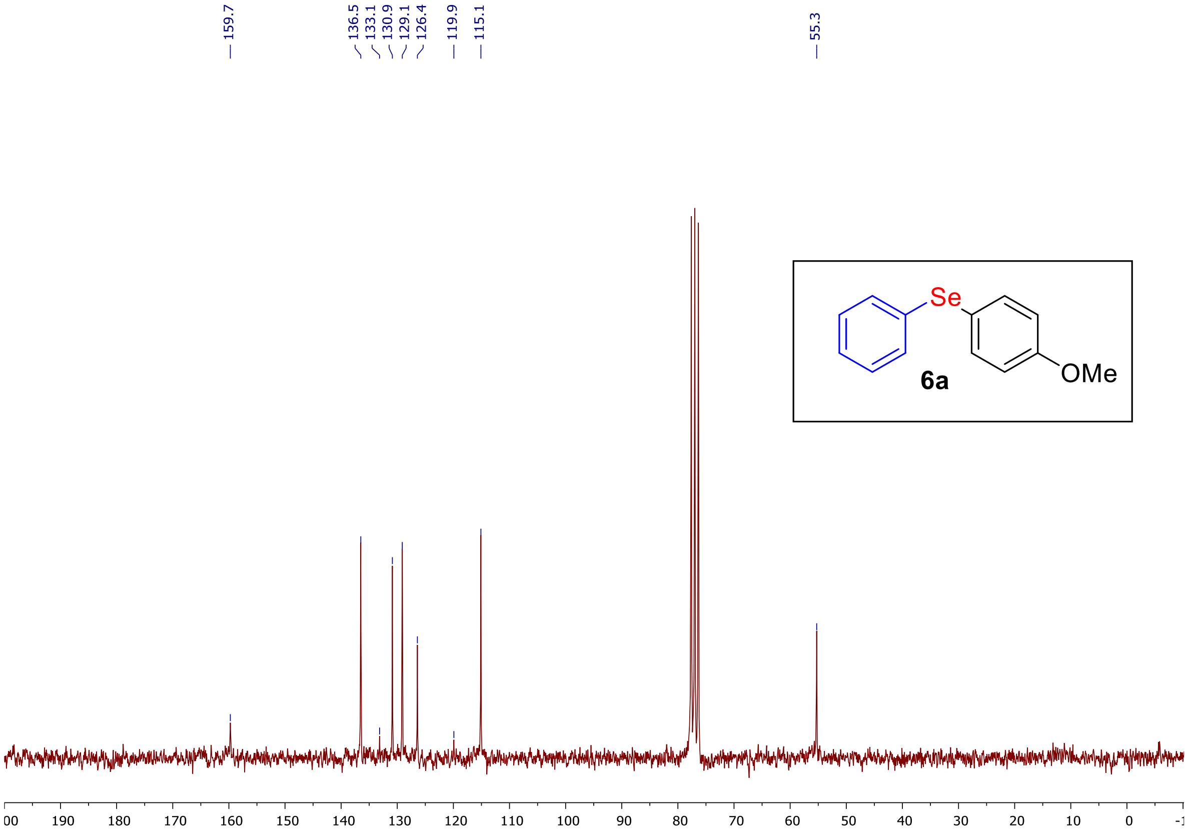

^{13}C NMR spectrum of compound **6a** in $CDCl_3$ (50 MHz)

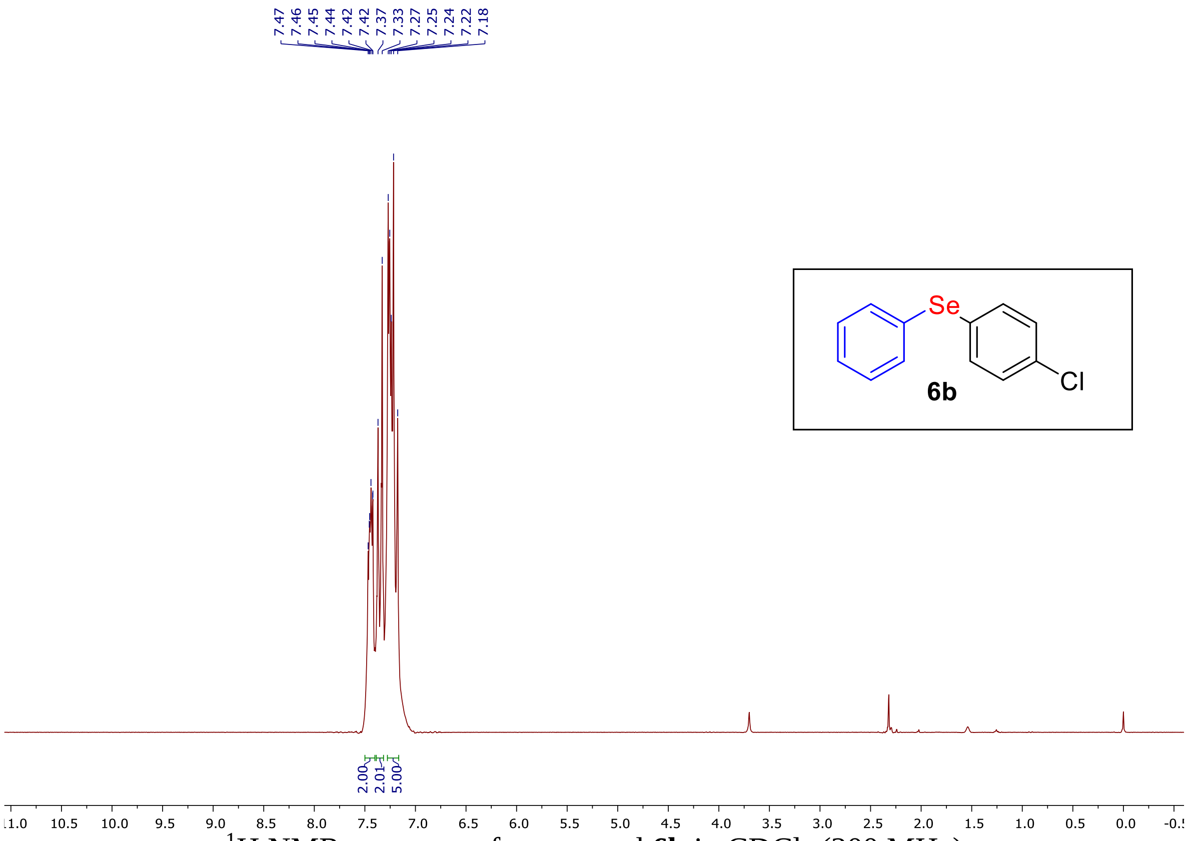

^{1}H NMR spectrum of compound **6b** in $CDCl_3$ (200 MHz)

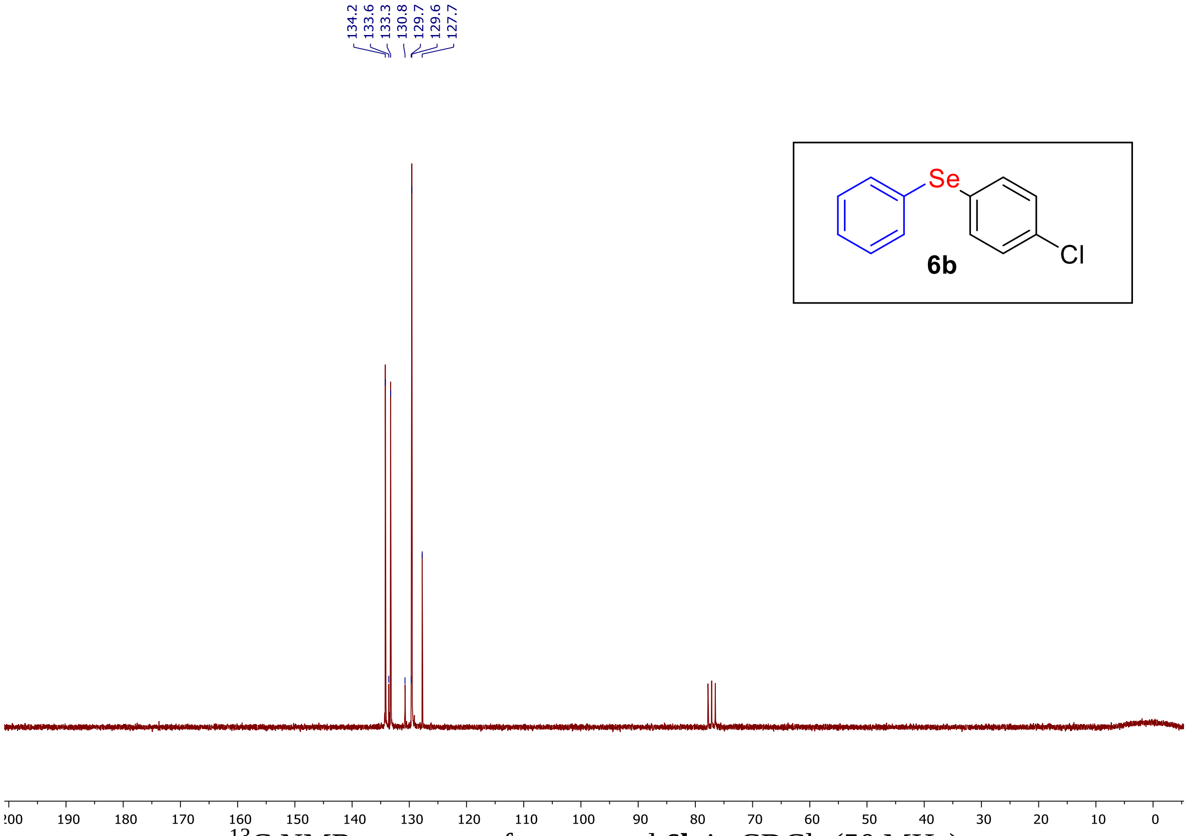

^{13}C NMR spectrum of compound **6b** in $CDCl_3$ (50 MHz)

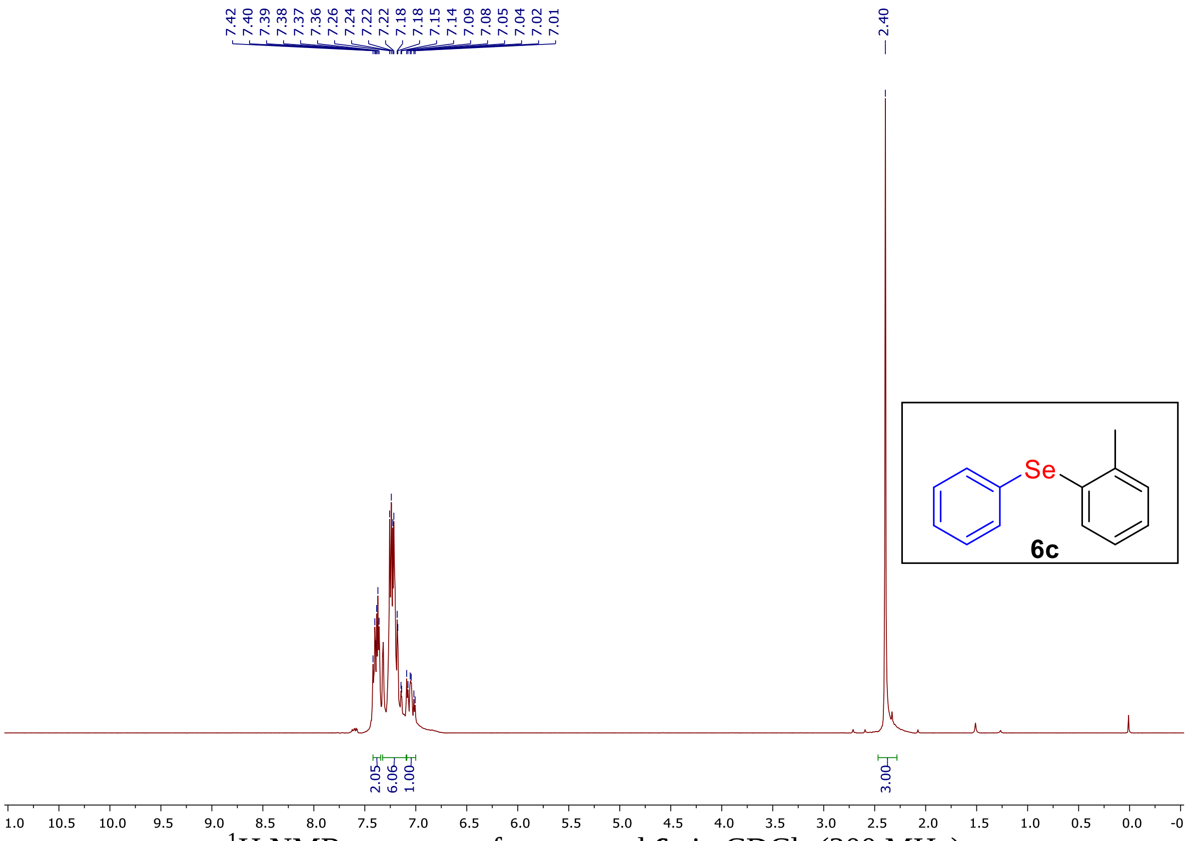

^{1}H NMR spectrum of compound **6c** in $CDCl_3$ (200 MHz)

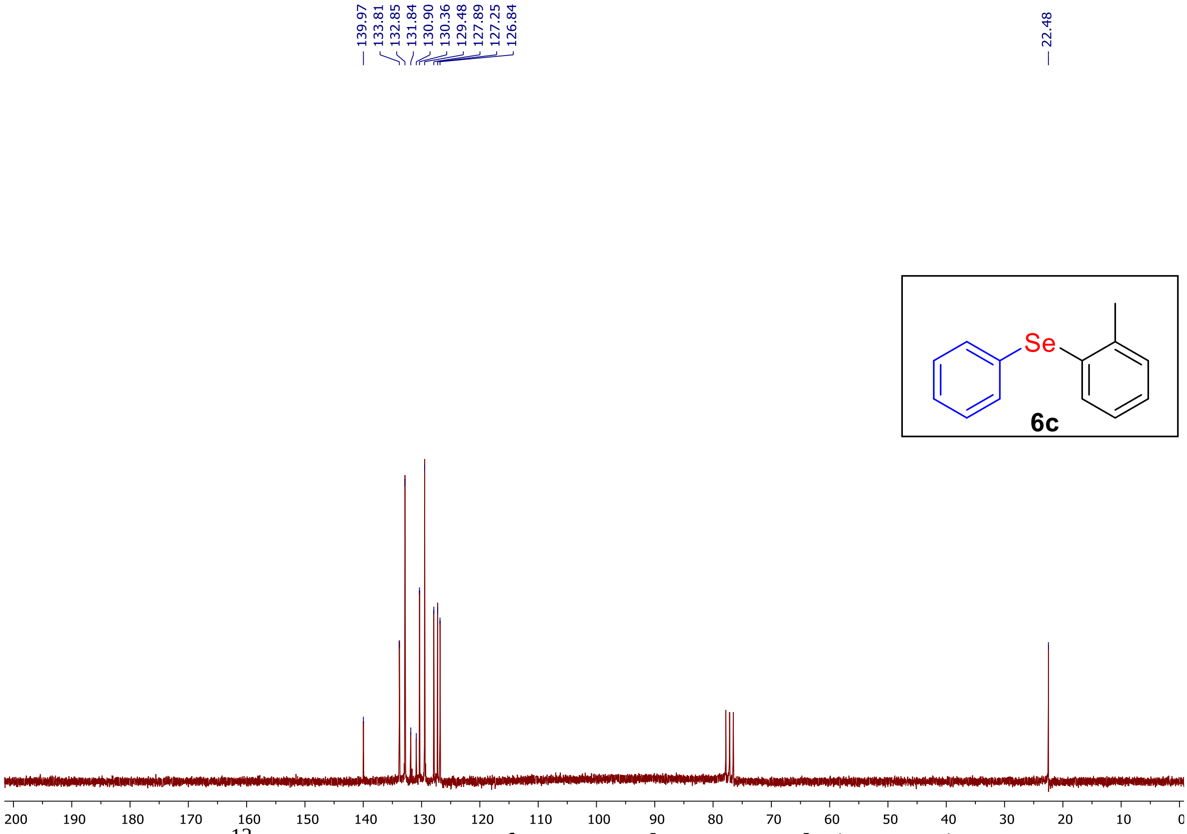

^{13}C NMR spectrum of compound **6c** in $CDCl_3$ (50 MHz)

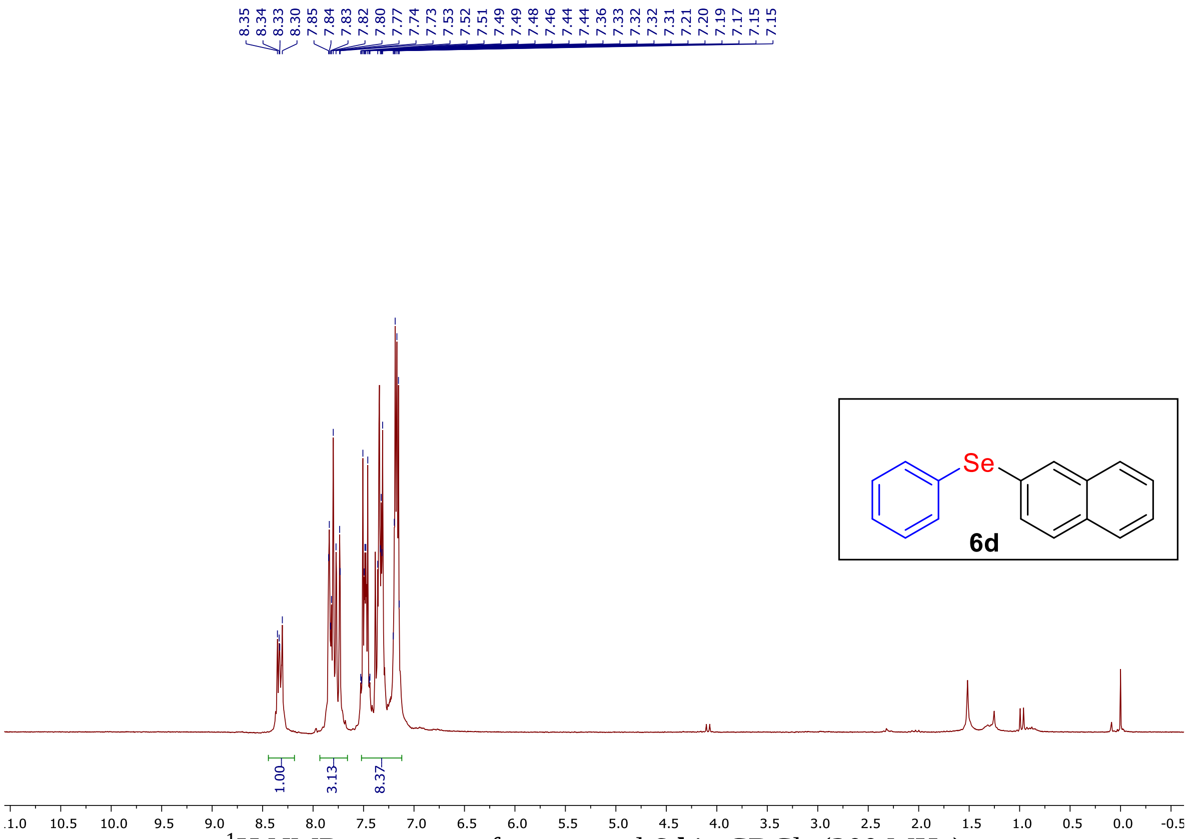

^{1}H NMR spectrum of compound **6d** in $CDCl_3$ (200 MHz)

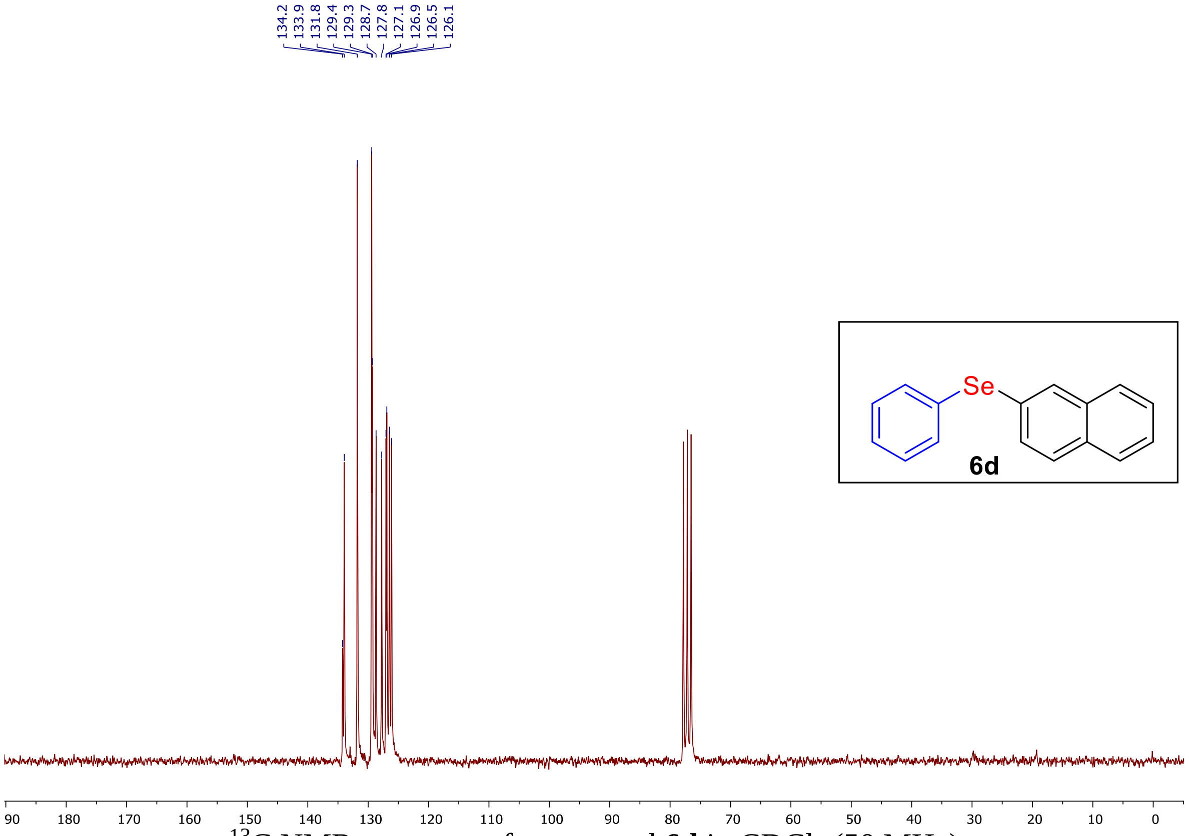

^{13}C NMR spectrum of compound **6d** in $CDCl_3$ (50 MHz)

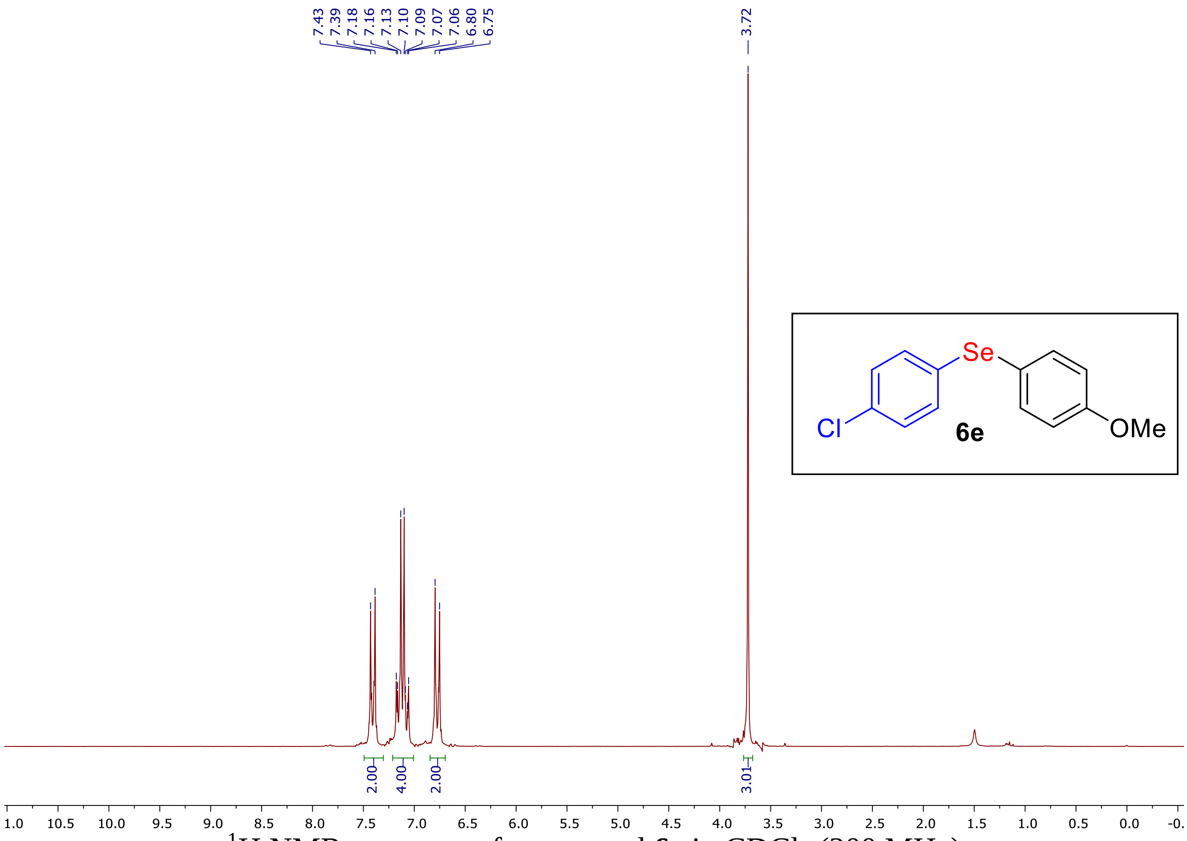

^{1}H NMR spectrum of compound **6e** in $CDCl_3$ (200 MHz)

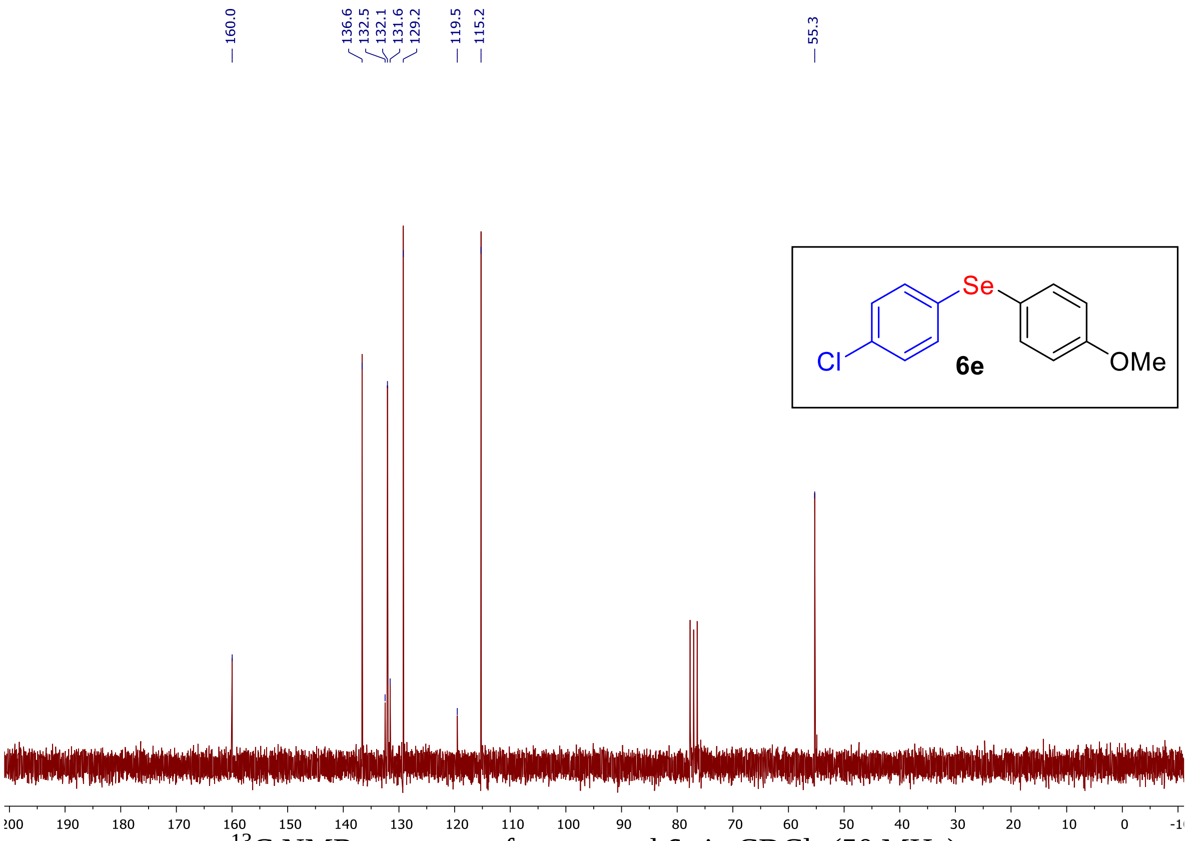

^{13}C NMR spectrum of compound **6e** in $CDCl_3$ (50 MHz)

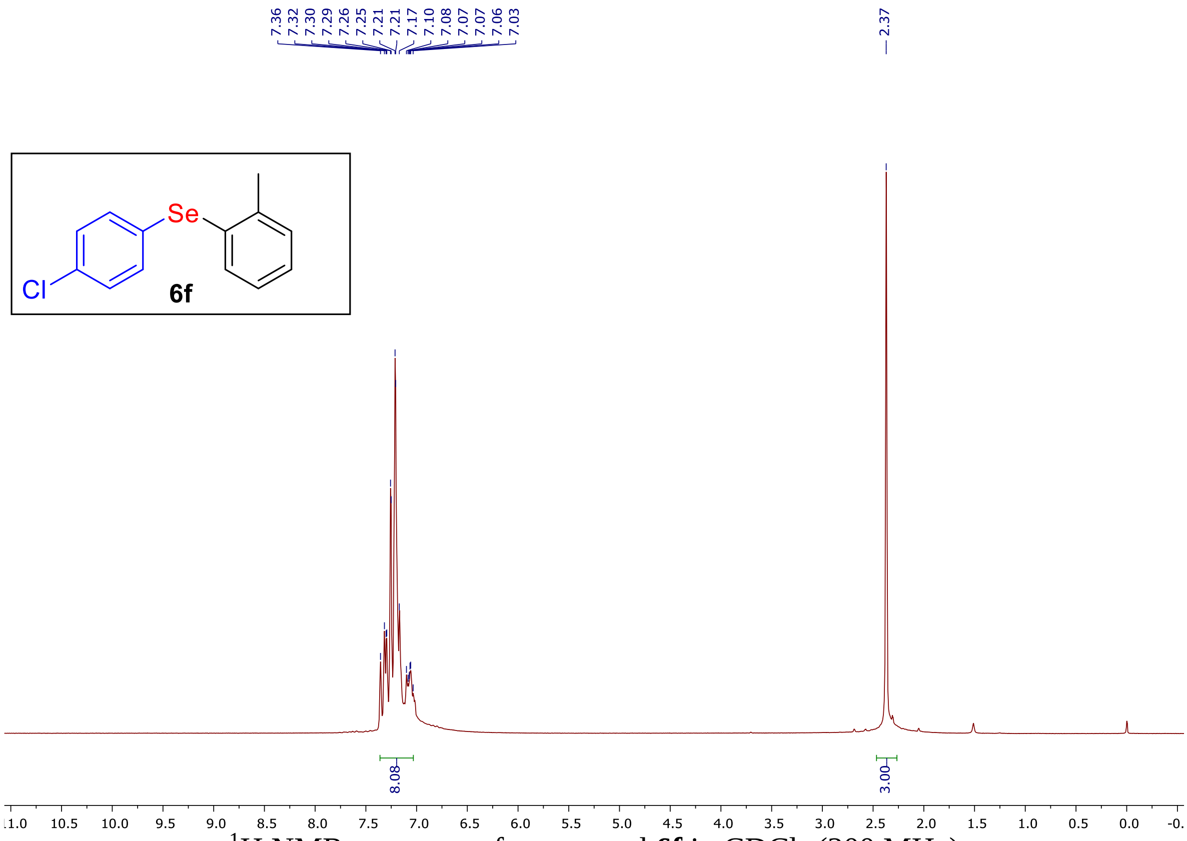

^{1}H NMR spectrum of compound **6f** in $CDCl_3$ (200 MHz)

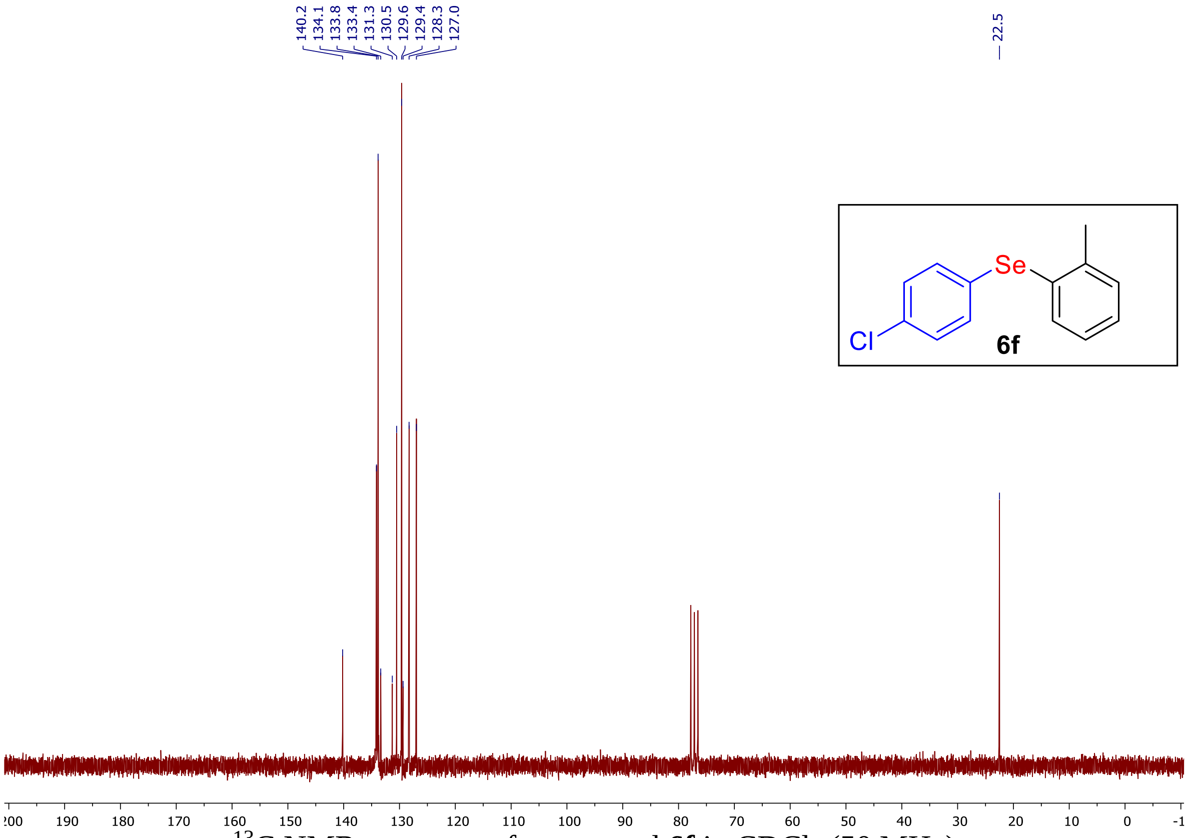

^{13}C NMR spectrum of compound **6f** in $CDCl_3$ (50 MHz)

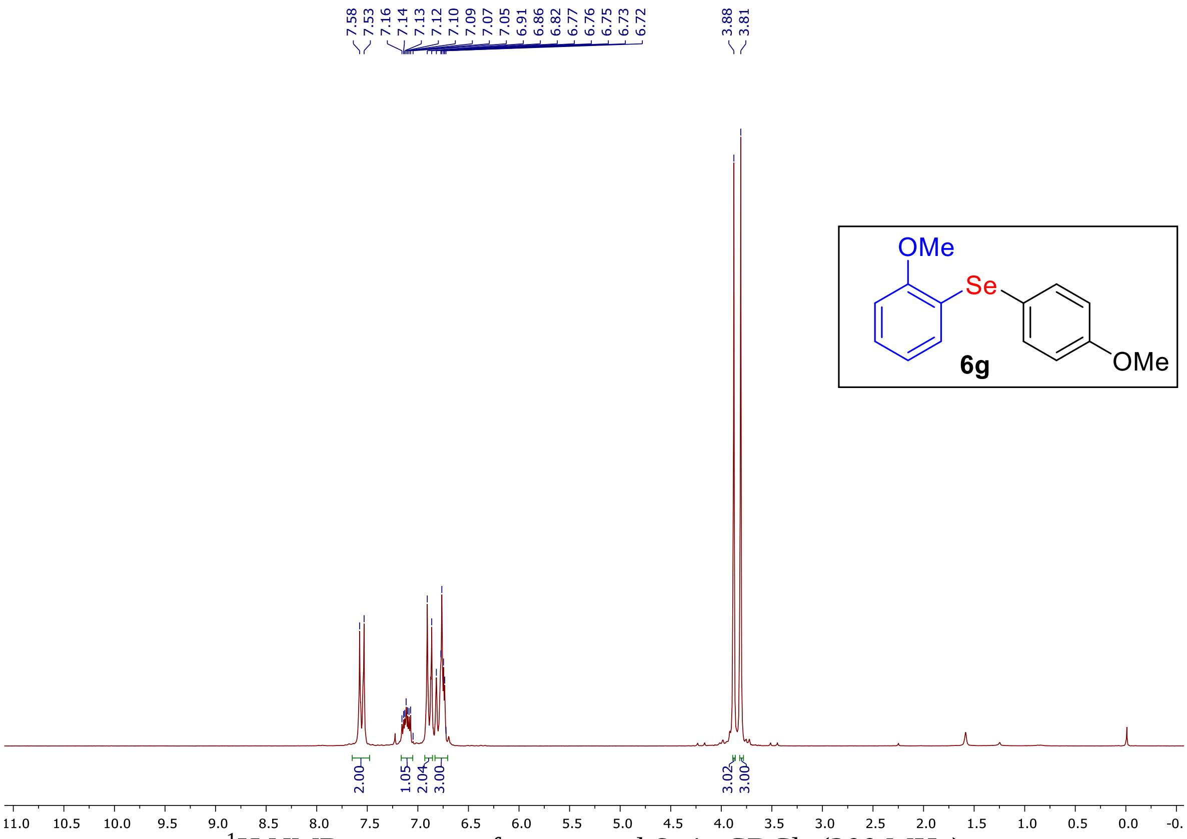

^{1}H NMR spectrum of compound **6g** in $CDCl_3$ (200 MHz)

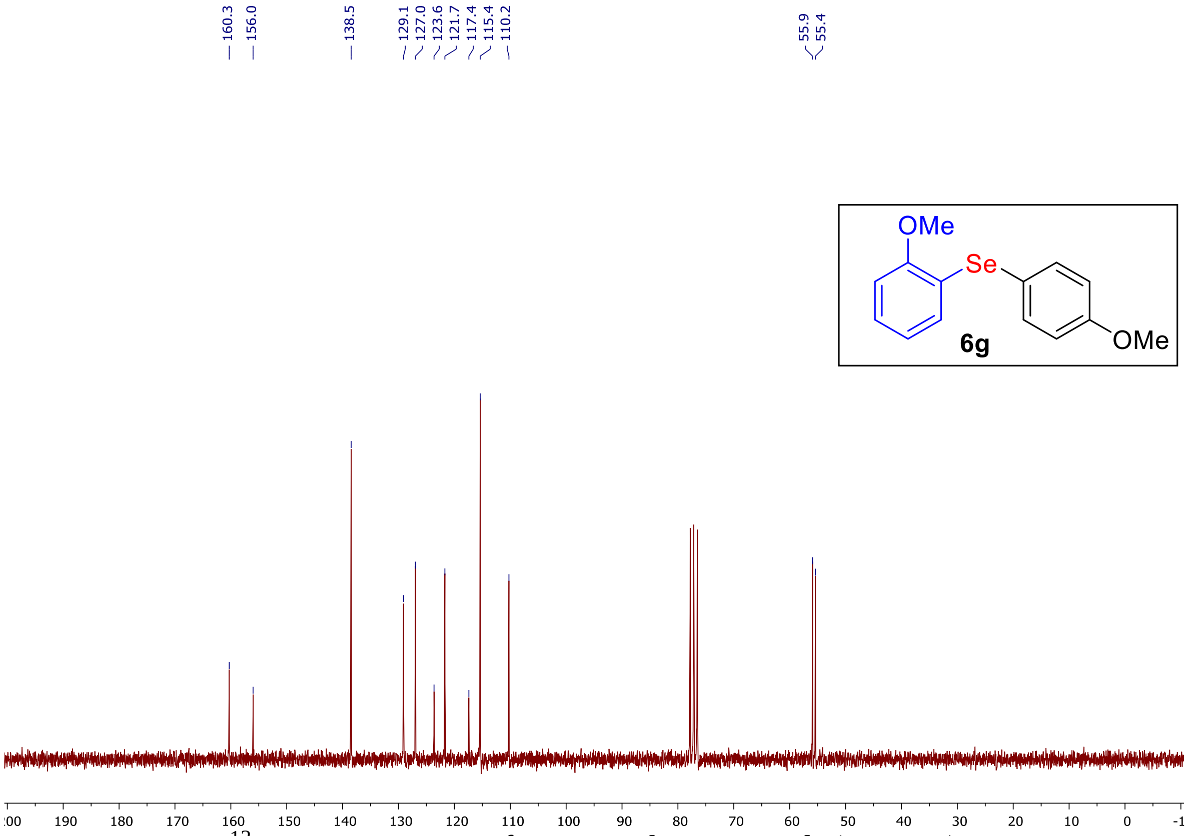

^{13}C NMR spectrum of compound **6g** in $CDCl_3$ (50 MHz)

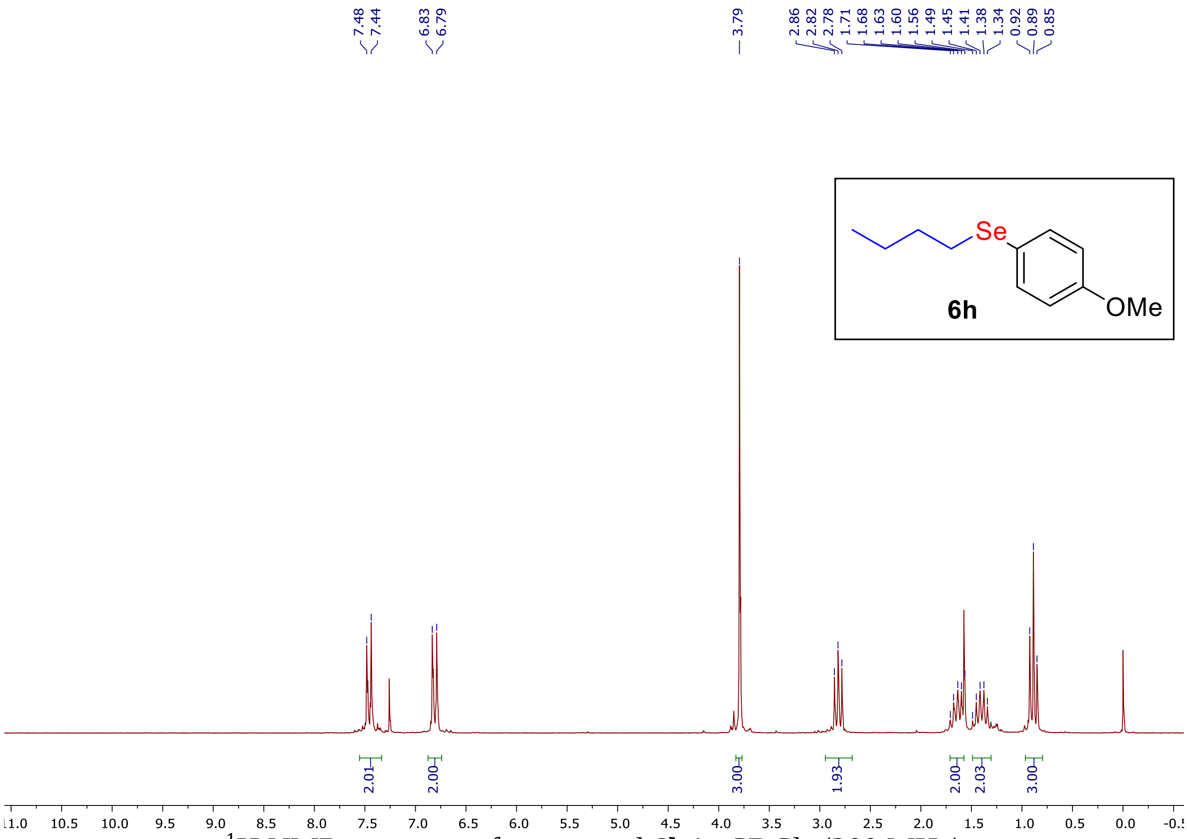

^{1}H NMR spectrum of compound **6h** in $CDCl_3$ (200 MHz)

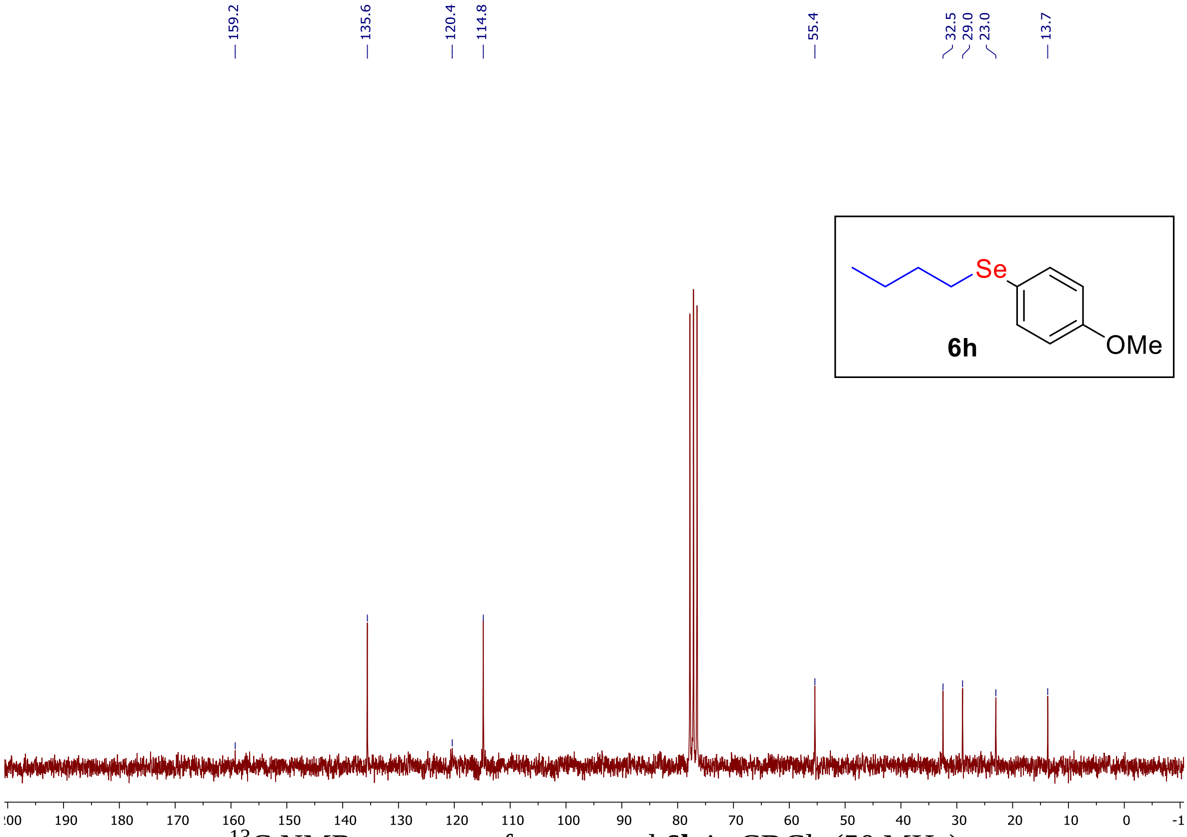

^{13}C NMR spectrum of compound **6h** in $CDCl_3$ (50 MHz)

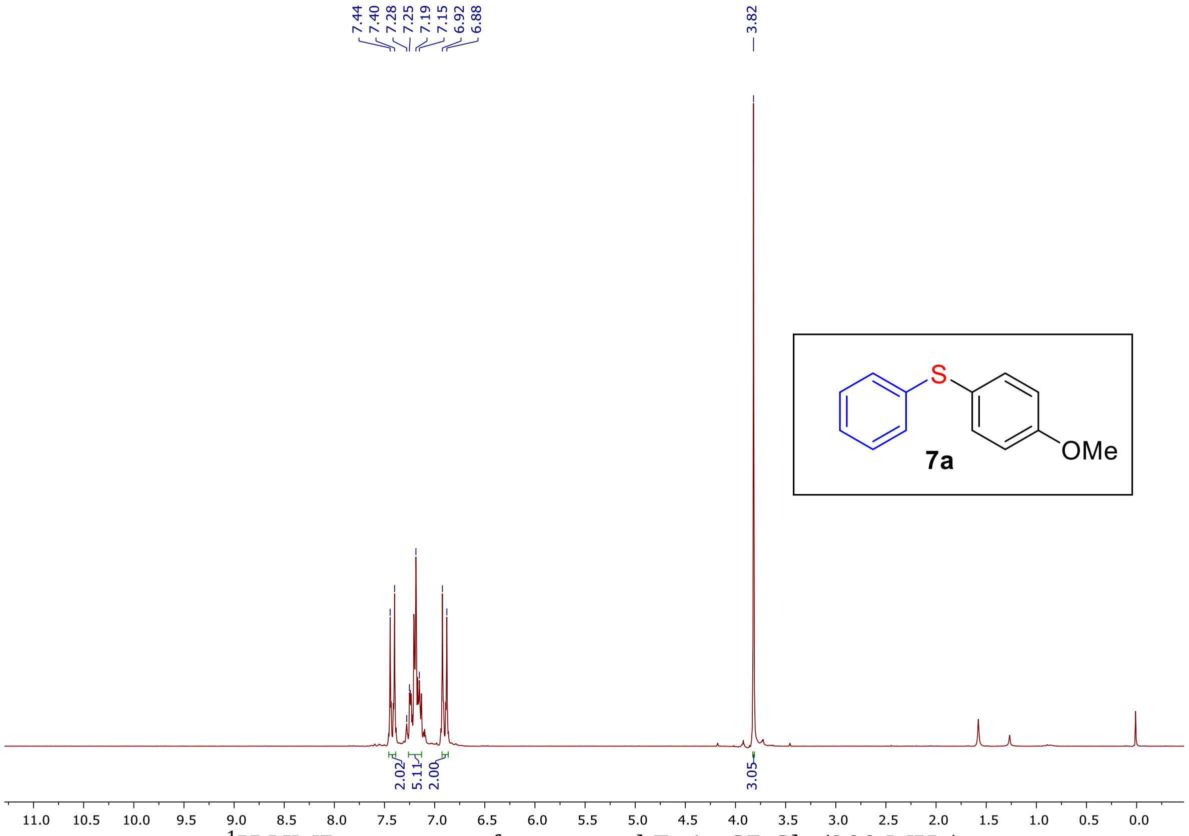

^{1}H NMR spectrum of compound **7a** in $CDCl_3$ (200 MHz)

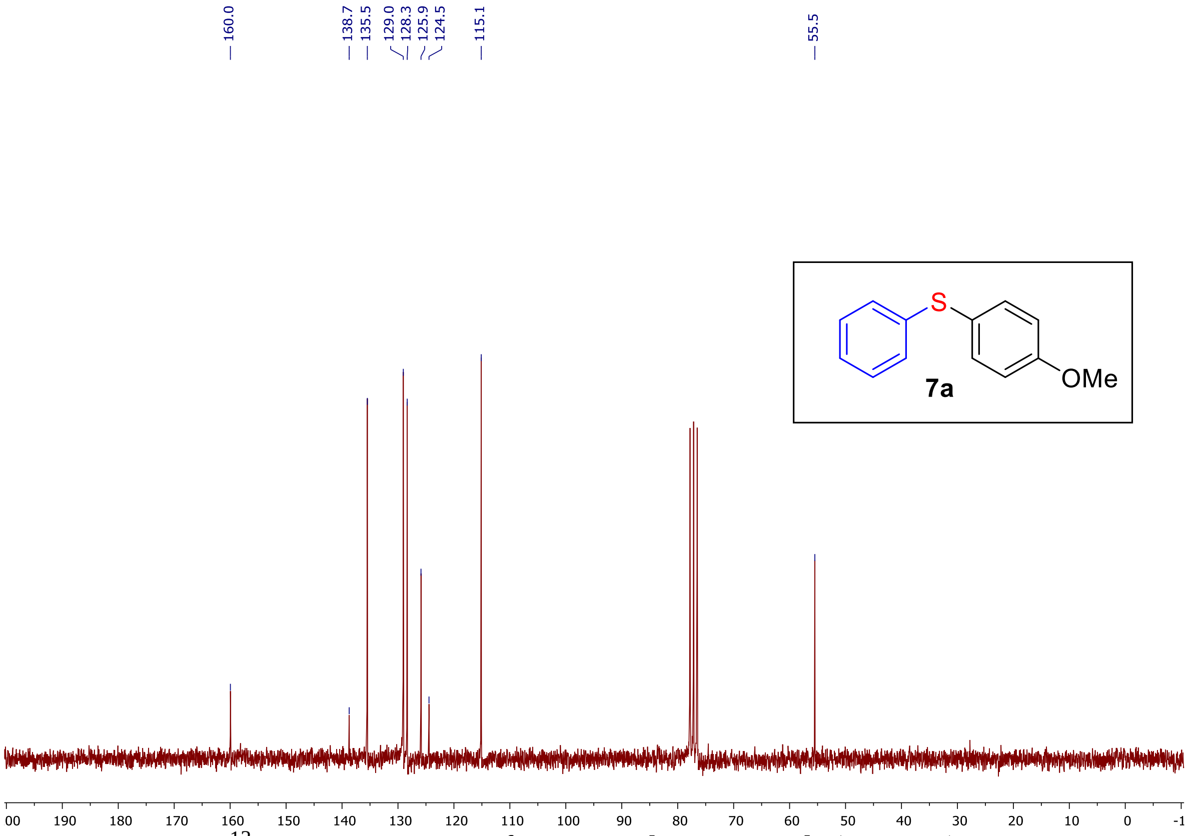

^{13}C NMR spectrum of compound **7a** in $CDCl_3$ (50 MHz)

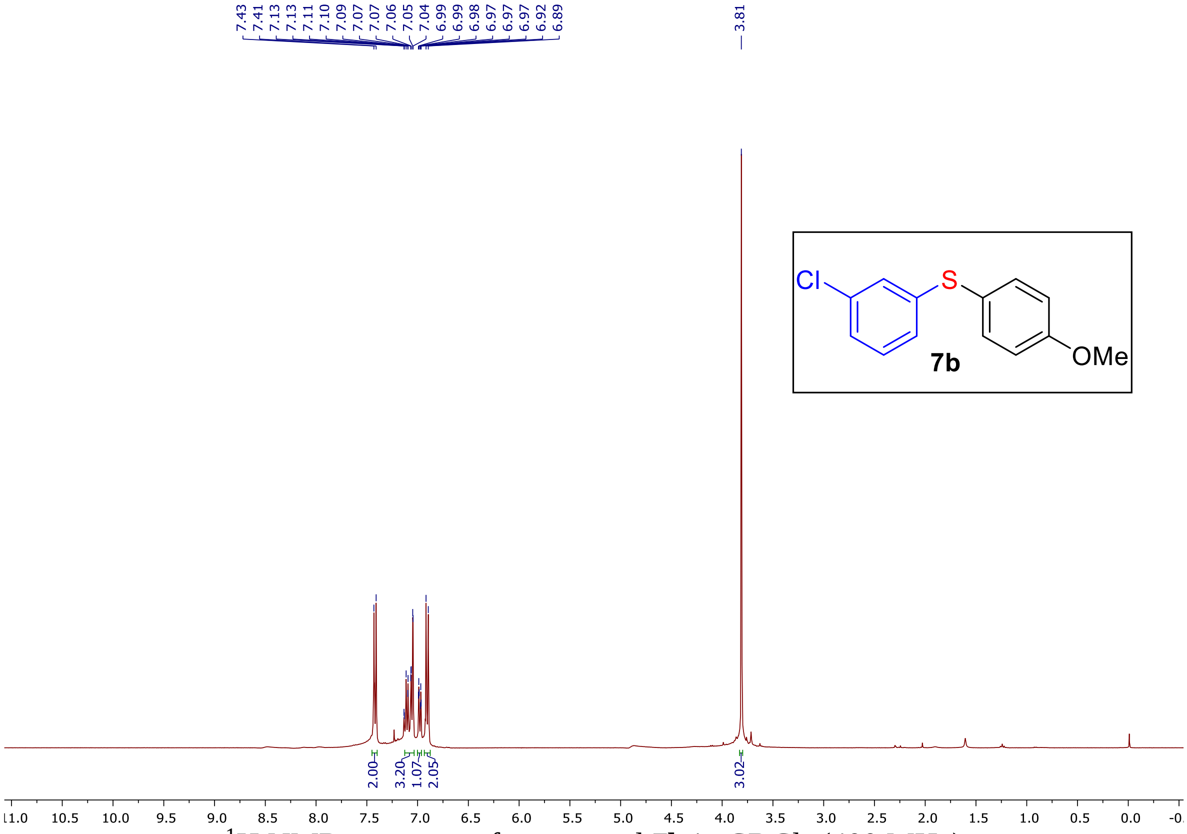

^{1}H NMR spectrum of compound **7b** in $CDCl_3$ (400 MHz)

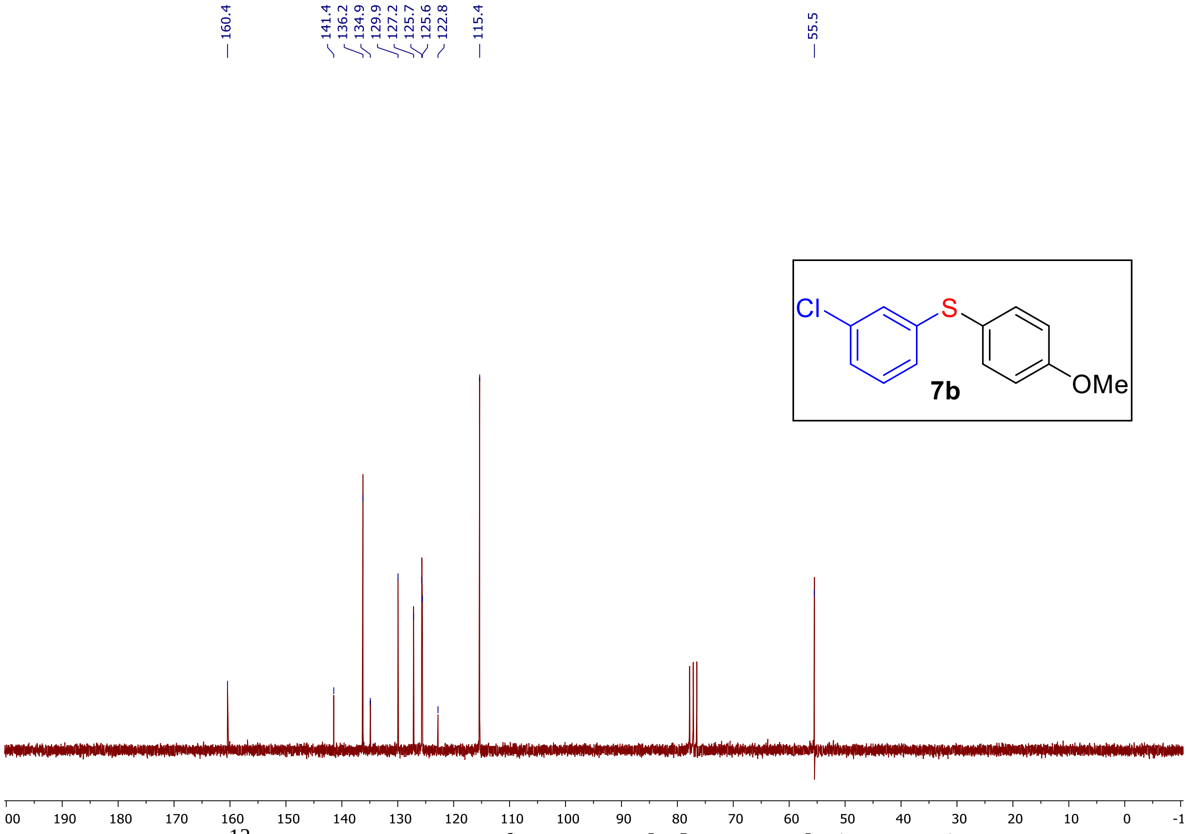

^{13}C NMR spectrum of compound **7b** in $CDCl_3$ (50 MHz)
